# α-Crystallin chaperone mimetic drugs inhibit lens γ-crystallin aggregation: Potential role for cataract prevention

**DOI:** 10.1016/j.jbc.2022.102417

**Published:** 2022-08-28

**Authors:** Sidra Islam, Michael T. Do, Brett S. Frank, Grant L. Hom, Samuel Wheeler, Hisashi Fujioka, Benlian Wang, Geeta Minocha, David R. Sell, Xingjun Fan, Kirsten J. Lampi, Vincent M. Monnier

**Affiliations:** 1 Department of Pathology and Biochemistry, Case Western Reserve University, Cleveland, Ohio, USA; 2 Department of Integrative Biosciences, Oregon Health & Sciences University, Portland, Oregon, USA; 3 Cryo-EM Core Facility, School of Medicine, Case Western Reserve University, Case Western Reserve University, Cleveland, Ohio, USA; 4 Center for Proteomics and Bioinformatics, Department of Nutrition, Case Western Reserve University, Cleveland, Ohio, USA; 5 Department of Cell Biology and Anatomy, Augusta University, Georgia, Georgia, USA; 6 Department of Biochemistry, Case Western Reserve University, Cleveland Ohio, USA

**Keywords:** fluorescence spectroscopy, oxidation, glycation, unfolding, diabetes, aging, drug repurposing, A, agaric acid, B, bixin, bis-ANS, bis-8-anilino-1-naphthalene sulfonic acid, C, closantel, D, dehydrogambogic acid, DLS, dynamic light scattering, DMSO, dimethyl sulfoxide, DTPA, diethylenetriaminepentaacetic acid, E, escin, F9S, phenylalanine to serine mutation at ninth position of WT mouse gamma S crystallin, G, gambogic acid, HγD, human gamma D, HγS, human gamma S, HSQC, heteronuclear single quantum coherence, M, chaulmoogric acid, MγS, mice gamma S, MW, molecular weight, PBS-T, PBS with 0.1% Tween-20, PDB, Protein Data Bank, MAC, mini-αA-crystallin chaperone, R14C, arginine to cysteine mutation at 14th position of WT human gamma D crystallin, R58H, arginine to histidine replacement at 58th position of WT human gamma D crystallin, S, sennoside A, T, tetradecyl sulfate sodium, V, avocadene, W43R, tryptophan to arginine mutation at 43rd position of WT human gamma D crystallin, X, hexachlorophene

## Abstract

Γ-Crystallins play a major role in age-related lens transparency. Their destabilization by mutations and physical chemical insults are associated with cataract formation. Therefore, drugs that increase their stability should have anticataract properties. To this end, we screened 2560 Federal Drug Agency–approved drugs and natural compounds for their ability to suppress or worsen H_2_O_2_ and/or heat-mediated aggregation of bovine γ-crystallins. The top two drugs, closantel (C), an antihelminthic drug, and gambogic acid (G), a xanthonoid, attenuated thermal-induced protein unfolding and aggregation as shown by turbidimetry fluorescence spectroscopy dynamic light scattering and electron microscopy of human or mouse recombinant crystallins. Furthermore, binding studies using fluorescence inhibition and hydrophobic pocket–binding molecule bis-8-anilino-1-naphthalene sulfonic acid revealed static binding of C and G to hydrophobic sites with medium-to-low affinity. Molecular docking to HγD and other γ-crystallins revealed two binding sites, one in the “NC pocket” (residues 50–150) of HγD and one spanning the “NC tail” (residues 56–61 to 168–174 in the C-terminal domain). Multiple binding sites overlap with those of the protective mini αA-crystallin chaperone MAC peptide. Mechanistic studies using bis-8-anilino-1-naphthalene sulfonic acid as a proxy drug showed that it bound to MAC sites, improved *T*_m_ of both H_2_O_2_ oxidized and native human gamma D, and suppressed turbidity of oxidized HγD, most likely by trapping exposed hydrophobic sites. The extent to which these drugs act as α-crystallin mimetics and reduce cataract progression remains to be demonstrated. This study provides initial insights into binding properties of C and G to γ-crystallins.

Cataract prevalence increases with age and affects more than 60% of US citizens over the age of 79. Globally, it is estimated that more than 150 million eyes have visions less than 6/60 because of cataract (1). Its incidence results in more than 18 million blinded individuals worldwide (2). While good vision is generally obtained with intraocular lens implant, up to 10% of individuals develop complications such as posterior subcapsular opacification (3). However, cataract is insidious because blurred vision constitutes a long prodromal period prior to cataract surgery that is associated with increased car accidents, depression, risk of falls, and mortality (4, 5, 6). Thus, there is an unmet pharmacological need as to the development of anticataract agents, whereby it has been estimated that delaying the onset of cataract by 10 years would halve the need for cataract surgery (7).

A number of mechanisms contribute to the extraordinary longevity and transparency of the human lens, which include a quasi-anoxic milieu and reducing environment linked to high glutathione levels and antioxidant defenses (8), the presence of UV filters (9), and above all, the unique composition and supramolecular organization of its structural proteins, the α-, β-, and γ-crystallins. The concentration of the latter reaches 400 mg/ml in the nuclear part of the lens (10), putting it at risk of spontaneous crystallization. This is however prevented by α-crystallins that are members of the small heat shock family of proteins, which act as molecular chaperones onto the βγ-crystallin family, thus shielding them for physical–chemical insults such as photo-oxidation and thermal stress (11, 12). With advancing age, however, there is a progressive breakdown of all defenses, and crystallins are increasingly deamidated (13), oxidized (14), glycated (15), and form covalent disulfide and nondisulfide crosslinks that contribute to their destabilization (16). Most notably, the chaperone activity of the α-crystallins is weakened because of insolubilization (17), unfolding (18), fragmentation, and generation of peptide fragments that enhance formation of light scattering aggregates (19).

In this work, we have focused on finding small molecules that might protect the gamma crystallins from destabilization and aggregation. These proteins are thought to play a particular role in the transparency of lens nucleus. They are small monomeric densely packed proteins with an N- and C-terminal domain each consisting of two Greek keys making them quite resistant to thermal and other stresses (20). When such stress is applied, the N-terminal domain tends to unfold first (21), a process that is enhanced with certain cataract-prone mutations, such as the W43R mutation (22). By now, more than 31 and 15 cataract-associated SNPs and mutations have been described in mouse and human γ-crystallin, respectively (23), and the list keeps growing (24). In the end, the pharmacological challenge is to find ways to prevent the formation of the light scattering aggregates. However, the types and mechanisms of aggregation are heterogenous and fall into at least three categories (25). The first consists of amyloid fibril formation that can be induced, for example, by exposing γD crystallins to low pH or photo-oxidative stress (26, 27). However, there is currently only limited evidence in support of amyloid fibril presence by EM in the aging human lens and cataract (M.J. Costello, personal communication, 2022) (28). In contrast, amorphous aggregates are probably dominant in both age-related cataract (29)as well as heat and UV-induced aggregation models (30). However, these amorphous aggregates may have multiple mechanisms of formation such as metal bridging (31, 32), domain swapping (33), and disulfide exchange (34). In addition, native protein aggregates may also occur in which certain mutant proteins fall out of solution and precipitate or even crystallize out without major structural changes, as reported for the R58H and R36S mutants of human gamma D (HγD) (35).

Based on the aforementioned understanding of the field, we have chosen to screen for compounds that can prevent a generic mixture of bovine gamma crystallins from forming amorphous aggregates upon exposure to thermal stress, with or without prior exposure to oxidant stress, followed by testing of the most active compounds against various recombinant native and mutant γ-crystallins.

## Results

### Screening for molecules that inhibit gamma crystallin aggregation

**Figure 1:**
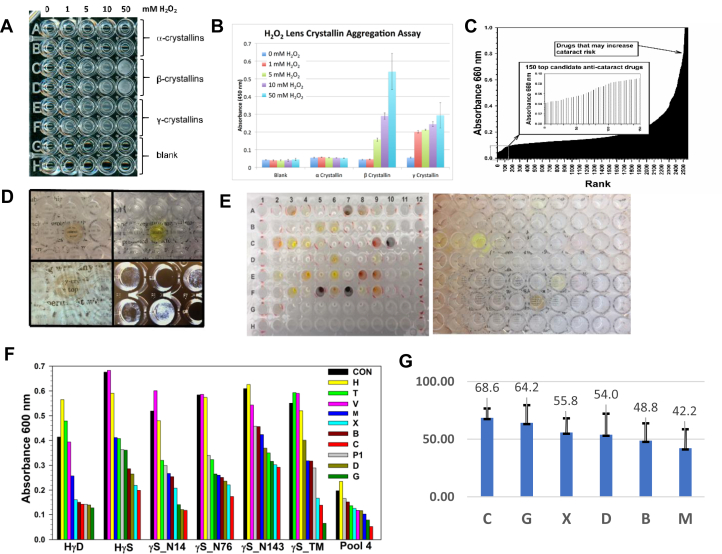
**Testing conditions for screening of drugs with potential anti-cataract activity.***A*, solutions of bovine lens crystallin fractions (2 mg/ml in 50 mM K_3_PO_4_ buffer) rich in β-crystallin and γ-crystallin become turbid when exposed to H_2_O_2_ at 37 °C up to 72 h. *B*, turbidity is quantified using absorbance at 450 or 600 nm. Note that α-crystallin-rich proteins are considerably resistant to heat denaturation. *C*, fitness plot ranking from highest to lowest for assessing turbidity inhibition potency of 2650 small molecules added at 500 μM concentration to gamma-crystallins exposed H_2_O_2_ (45 mM) at 37 °C for 24 h. The profile of the top most potent drugs is shown in the *inset*. Conceptually, compounds that suppress turbidity have anticataract activity, whereas compounds that increase it are cataractogenic. *D*, representative examples of turbidity suppression by three colored drugs allowing fine text read-through and visible protein aggregate suppression by dark field microscopy. *E*, a sublibrary of about 80 compounds (*left*) was retested at 150 μM concentration from which the best compounds were selected for further studies based on text read-through testing (*right*). *F*, to validate the initial screening results, top candidate compounds were screened for turbidity suppression of solutions of recombinant hCRYGD and hCRYGS, its deamidation mutants N14D, N76D, N143D, and the triple mutant TM. Drugs in 10% dimethyl sulfoxide (DMSO) were hematoporphyrin (H), tetra sodium sulfate (T), V (avocadene), chaulmoogric acid (M), hexachlorophene (X), bixin (B), closantel (C), gambogic acid (G), dihydrogambogic acid (D) *versus* bovine α-crystallin (pool 1) and 10% DMSO as negative control (CON). *G*, bar graph shows the mean aggregation suppression score of the top six most potent drugs C, G, X, D, B, and M based on a composite of all screening data in bovine, HγD and HγS crystallins (*F*) and the extended data in *B*. Drugs C (closantel) and G (gambogic acid) emerged as the consistently most effective aggregation suppressors from 2650 compounds tested. HγD, human gamma D; HγS, human gamma S.

For the initial screen, a microtiter plate assay was developed in which bovine crystallins (2 mg/ml of 50 mM K_3_PO_4_ buffer, pH 7.2 [buffer A]) were exposed to H_2_O_2_ at varying concentrations (1–50 mM) for 12 to 36 h, upon which turbidity developed (at 50 mM H_2_O_2_ concentration) in both β- and γ-crystallins (Fig. 1, *A* and *B*). About 2560 compounds from the Microsource Discovery Library were initially tested at 500 μM in 5% dimethyl sulfoxide (DMSO) in buffer A for their ability to prevent H_2_O_2_ and heat mediated aggregation of purified bovine gamma crystallins (2 mg/ml) over 12 to 48 h. This was determined by turbidimetry at 600 nm (Fig. 1*C*) and confirmed using visual assessment of solution transparency over a fine text as well as the presence of cloudy aggregates visible by dark field microscopy (Fig. 1*D*). The drug:protein stoichiometric ratio was about 5:1. The wavelength of 600 nm was chosen to minimize interference by colored compounds, many of which absorb below 500 nm (Fig. 1*E*). A fitness plot was generated that resulted in a narrower choice of 135 potential anticataract drugs and 241 compounds with marked destabilizing and therefore potential cataractogenic effect (Fig. 1*C*). A table identifying all compounds with antiaggregation (n = 135) and proaggregation (n = 241) activity from Figure 1*C* is included (Table S10).

Candidate compounds were rescreened using thermal denaturation upon heating at 72 °C for 20 min according to Chen *et al.* (36) in order to select for compounds with chaperone rather than antioxidant activity since a large number of the latter already exist in the cataract field (37). A subscreen involving 80 compounds (Fig. 1*E*) tested at 150 μM concentration resulted in nine compounds with thermal denaturation inhibitory activity. These include hematoporphyrin (H), tetrasodium sulfate (T), avocadene (V), chaulmoogric acid (M), hexachlorophene (X), bixin (B), closantel (C), gambogic acid (G), and dihydrogambogic acid (D).

For refinement of the aforementioned screen, these compounds were tested at concentrations varying from 50 to 500 μM and 1.5 mg/ml concentration of recombinant HγD, human gamma S (HγS), its deamidation mutants γS_N14, γS_N76, γS_N143 and its triple mutant γSTM (Fig. 1*F*). In addition, other drugs and compounds that tested positive in the initial screen were also included, such as escin (E), hematein (I), citric acid (CA), docusate sodium (DS), and sennoside A (S) (Fig. S1*B*). Table 1 provides structural and other information about the most active candidate drugs, and Table 2 provides a summary of most active candidate drugs against thermal aggregation of γ-crystallins and their deamidation mutants. In these experiments, pool 1 (purified high–molecular weight [MW] bovine α-crystallins) was used as a positive control and found to potently inhibit both pool 4 protein (bovine γ-crystalline-rich fraction) and HγD aggregation, as similarly reported by multiple authors. For a review, see the study by Roskamp *et al.* (38). Using the data generated in Figures 1*F* and S1*B*, mean inhibitory activity was calculated for all drugs across all γ-crystallins tested in order to determine the top six most active aggregation inhibitors. Based on 69, 64, 56, 54, 49, and 42% inhibition of γ-crystallin aggregation against thermal denaturation by drugs C, G, X, D, B, and M respectively, ranking was assigned in the order C > G > X > D > B > M with C being strongest and M weakest inhibitors of thermal aggregation (Fig. 1*G* and Table S1). Thus, drug C and drug G emerged as the strongest antidenaturation/aggregation and potential anticataract drugs. For the mechanistic studies below these two compounds together with either drug M, chosen mostly as negative control, or the entire set of six compounds were used for comparison purpose, whereby the words “drug” or “compound” are used interchangeably in the studies later.

**Table 1:** Summary of tested drugs displaying antiaggregation activity toward bovine γ-crystallin-rich proteins exposed to oxidant stress for 24 h and/or thermal stress at 72 °C for 20 min

Compound name	MW (g)	Usage	Properties	Antiaggregation activity	Provenience, appearance
Bixin (drug B)	394.5	Food color	Antineoplastic	Medium heat shock inhibitor	Orange colored solid
Closantel (drug C) Two enantiomers C(R) and C(S)	663.1	Clinical drug	Antihelminthic	Medium strong dual inhibitor	Synthetic, clear solid, colorless
Chaulmoogric acid (drug M)	280.4	Experimental drug	Antibacterial, antileprotic	Dual heat shock inhibitor + antioxidant	Natural product, colorless, oily
Gambogic acid (drug G)	628.7	Experimental drug	Anti-inflammatory, anticancer, multiple actions	Strong heat shock inhibitor	Natural product, yellow solid
Dihydrogambogic (drug D) Acid (reduced G)	630.8	Experimental drug	Undetermined activity	Strong heat shock inhibitor	Modified natural product, solid
Hexachlorophene (drug X)	406.9	Clinical drug	Topical antimicrobial	Medium heat shock inhibitor	Synthetic, solid, colorless

**Table 2:** Summary of the most active candidate drugs with antiaggregation activity against various γ-crystallins exposed to heating at 72 °C for 20 min

Crystallin type	Drug codes in order of efficacy	Stoichiometric drug:protein ratio	% Suppression
Bovine γ-crystallins^a^			
1.	CGMDVX	1.7:1	60/48/40/40/36/30
2.	GCXBVD	1.7:1	75/73/55/54/53/44
HγD^a^			
1.	GDCBXM	1.7:1	69/66/65/63/61/38
2.	DGCMBX	1.7:1	78/75/73/73/60/47
HγS	CXDBGT	1.7:1	71/68/61/60/46/40
HγS_N14D	CDGXBM	1.7:1	77/76/73/60/51/48
HγS_N76D	CXDBMG	1.7:1	70/60/59/57/55/55
HγS_N143D	CXGTDM	1.7:1	52/50/48/42/30/25
HγS_N14/76/143D	GCXBMD	1.7:1	89/76/71/46/46/32

The proteins were tested at 75 μM concentration (1.5 mg/ml of 50 mM K^+^PO_4_^3−^, pH 7.2) incubated for 20 min at 72 °C with 125 μM or less inhibitor concentration. This corresponds to 1.7:1 stoichiometric drug:protein ratio. For drug codes, refer to Table 1.

a Denotes results from separate experiments.

### Drug C and G inhibit thermal unfolding and aggregation of human recombinant γD crystallin and its mutants

**Figure 2:**
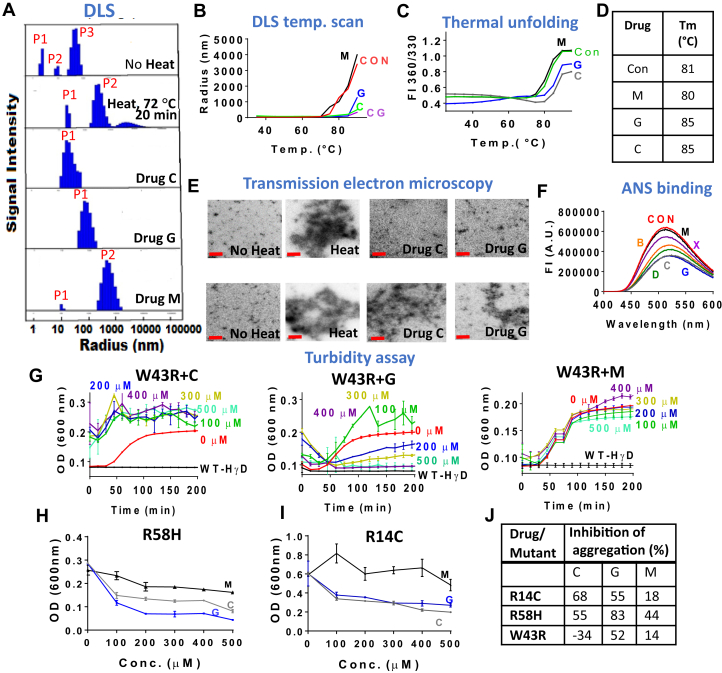
**Inhibitory effect of closantel (C) and gambogic acid (G) against heat-induced aggregation of HγD crystallin and its mutants.** Chaulmoogric acid (drug M) served as negative control. *A*, dynamic light scattering spectra of thermally stressed (72 °C, 20 min) WT-HγD crystallin (50 μM in 50 mM K_3_PO_4_ buffer, pH 7.2) alone and with drugs C, G, and M (400 μM in dimethyl sulfoxide [DMSO]). *B*, DLS temperature scan of 50 μM of WT-HγD samples with and without drug C, G, and equimolar CG together. Samples were heated from 30 °C to 90 °C with 1 °C/min rise in temperature. *C*, thermal unfolding study of 10 μM WT-HγD crystallin with or without 100 μM of drug C, G, and M in DMSO. *D*, table showing *T*_m_ of WT-HγD alone and with drugs C, G, and M. *E*, transmission electron micrographs of WT-HγD (50 μM) heated with and without drugs C and G (400 μM). Two different images are presented for each condition of the same samples. The *red bars* indicate 200 nm scale. *F*, bis-ANS binding study of 10 μM WT-HγD with and without 100 μM of drugs (C, G, X, D, B, and M). *G*, effects of drugs C, G, and M (0–500 μM) on turbidity assay (kinetic study at 42 °C) of W43R mutant (43 μM in 50 mM K_3_PO_4_ buffer) *versus* WT-HγD. The readings were taken at an interval of every 10 min for a total of 200 min. End-point turbidity assay (thermal stress at 72 °C for 20 min) of R58H (*H*) and R14C (*I*) mutants of WT-HγD (50 mM K_3_PO_4_ buffer, 5 mM DTT) at different drug concentrations of C, G, and M. *J*, table showing percent inhibition of aggregation of mutants by 500 μM of the drugs (see Experimental procedures section for details). Each experiment was done in triplicates, and the results represent average ± std dev. of three individual readings. bis-ANS, bis-8-anilino-1-naphthalene sulfonic acid; DLS, dynamic light scattering. HγD, human gamma D.

His-tag free recombinant HγD crystallin produced in *Escherichia coli* using pET3d or pET17b expression vector was used to investigate the effects of compounds C and G against thermal-induced unfolding and aggregate formation. The antiaggregation potency of drug C and G was reconfirmed by dynamic light scattering (DLS) (Fig. 2*A*). Drug C and G showed excellent inhibition against aggregate formation as revealed by radius of aggregates of samples with and without drugs. There is a decrease in the size of aggregates from as high as 7261 nm without drugs to 30.5 nm with drug C and 84.9 nm with drug G. Drug M has moderate activity with mean aggregate size of 569 nm radius (Table S2). It should be noted that in comparison to the radius of unheated HγD crystallin (2.3 nm, 99%), the radius size was slightly increased with drug C (30.5) and G (84.9), suggesting that these drugs tend to prevent formation of larger size aggregates better than small ones. A DLS temperature scan from 30 to 90 °C confirmed the results with a hint that equimolar amounts of drug C and G combination are equally effective as either of them alone (Fig. 2*B*). Furthermore, we investigated whether these drugs can prevent thermal unfolding of HγD (Fig. 2*C*). The fluorescence ratio at 360 nm over 330 nm was used to determine the *T*_m_. It is the temperature at which 50% of the protein unfolds. The *T*_m_ of HγD was found to be elevated by 4 °C, that is, from 81 °C for the control to 85 °C for both C- and G-treated protein (Fig. 2*D*). This suggests that these drugs are tentatively stabilizing the tertiary structure of protein and resisting thermal unfolding to a significant extent. Drug M showed no resistance to unfolding of HγD.

The impact of drugs C and G on the morphology of the aggregates at 72 °C was assessed by transmission electron microscopy. Amorphous aggregates, similar to those previously reported in various mutant proteins (39), were detected to various extents in all images. No amyloid fibers were found at 100 nm resolution (images available upon request). Using dark field microscopy, granular precipitates observed at 10 min (Fig. 2*E*) formed thick fibers at 30 min in the absence of drugs (see later, images available upon request). Bis-8-anilino-1-naphthalene sulfonic acid (bis-ANS)–binding studies showed that compounds C and G were most efficacious in competing with bis-ANS fluorescence, an indicator of hydrophobic surfaces in HγD (40) (Fig. 2*F*). Table S3 provides details about percent inhibition in binding of bis-ANS by our selected drugs. The order in which these compounds suppress bis-ANS fluorescence matches the order of their antiaggregation activity.

**Figure 3:**
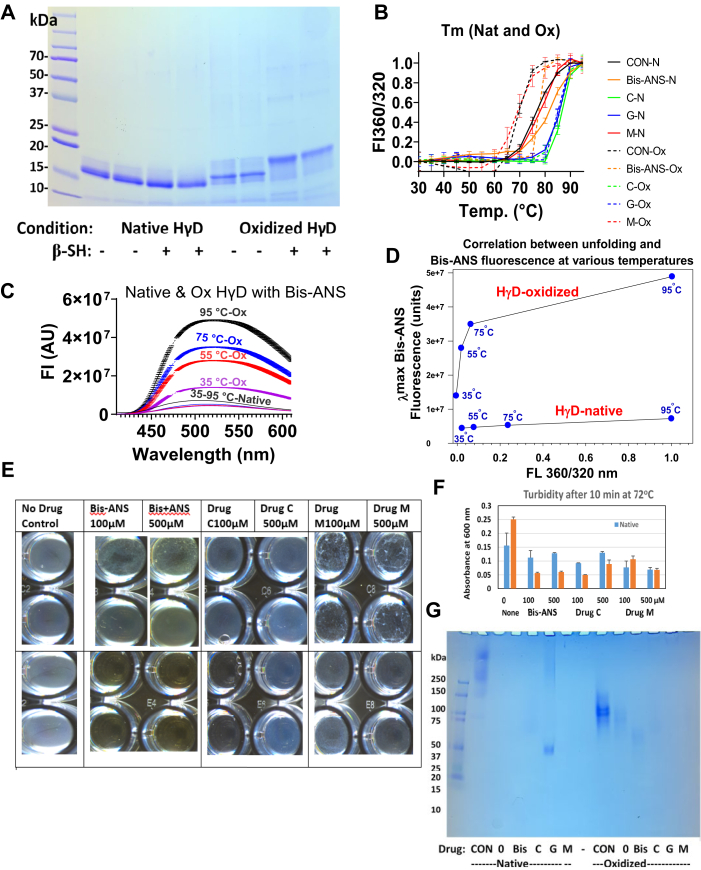
**Comparative studies of native and oxidized HyD.***A*, SDS gel of native and oxidized HγG with and without reduction with β-mercaptoethanol showing no effect of reduction on the native form of HyD but upward migration shift of oxidized HγD. *B*, comparative effects of drugs C, G, and M on *T*_m_ of native and oxidized HyD showing HyD-ox unfolds at lower temperature than native HyD with both drugs C and G, as well as bis-ANS, except M, improving the *T*_m_ of both proteins. *C*, bis-ANS fluorescence of native HγD barely increases with thermal stress, whereas marked increase is noted with the oxidized protein. *D*, correlation between unfolding based on 360/320 nm fluorescence ratio and bis-ANS fluorescence shows that bis-ANS fluorescence dramatically increases with moderate unfolding of HγD-ox, whereas relatively little bis-ANS binding is observed during unfolding of native HγD. *E* and *F*, effect of 100 and 500 μM bis-ANS and drugs C and M on absorbance at 600 nm (*F*) and dark field microscopic images (*E*) of native and oxidized HγD (1.4 mg/ml with DTPA) incubated for 10 min at 72 °C. The images of the microtiter wells reveal intense milky appearance of the control sample that was strongly suppressed by both 100 and 500 μM bis-ANS, 100 μM drug C, and 500 μM drug M. Note the presence of coalesced insoluble aggregates in the native protein incubated with drug M suggesting caution with the interpretation of turbidity data without morphological data. *G*, native PAGE of native and oxidized nonheated HγD protein (CON) and protein heated at 72 °C for 10 min without (0) or with 100 μM bis-ANS, C, G, and M drugs reveals no protein stain, likely because of blocking of Coomassie binding sites by the drugs except for drug G. bis-ANS, bis-8-anilino-1-naphthalene sulfonic acid; DTPA, diethylenetriaminepentaacetic acid; HγD, human gamma D.

The ability of the drugs to stabilize mutants of HγD was then studied for W43R, R14C, and R58H (Fig. 2,*G*–*I*). These mutants were generated in our laboratory by site-directed mutagenesis using the primers listed in Table S4. After confirmation of the mutations by Sanger sequencing, the proteins were expressed, purified, and identified using SDS-PAGE (Fig. S2*A*) and Western blot (Fig. S2*B*), respectively. W43R is a very unstable mutant of HγD that is associated with autosomal dominant congenital cataract (41). Among all four tryptophan residues, the one at position 43 is the most critical for its stability (22). Drug G (400 and 500 μM), though not drug C, protected the mutant against aggregation when kept at 42 °C for 200 min. Drug C at all concentrations worsened the aggregation of this mutant (−34%, Fig. 2*J*), whereas drug M showed no effect at any of the concentrations (Fig. 2*G*). R14C is a well-studied mutant that is associated with juvenile onset hereditary cataract (42). Because of the presence of an additional cysteine residue at position 14, it forms intermolecular disulfide crosslinked aggregates at physiological temperature and pH. Hence, to keep this protein stable, we added 5 mM DTT to the buffer. End-point turbidity assay (72 °C, 20 min) shows 68% prevention of thermal aggregation by drug C and 55% by drug G (Fig. 2,*I* and *J*). It should be noted that without DTT, the mutant was unstable even in the presence of drugs ruling out the possibility of using them to prevent the formation of disulfide linkages (data not shown). Drugs C and G were also found to protect R58H-HγD mutant (Fig. 2, *H* and *J*), which is associated with aceuliform cataract (43), against thermal aggregation (72 °C, 20 min) by 55 and 83%, respectively (Fig. 2*J*). The percent aggregation inhibition of the mutants of HγD by 500 μM of the drugs at the end point of turbidity assay is shown in Figure 2*J*. Upon heating HγD crystallin at 72 °C for 20 min, little change was observed in the far- and near-UV CD spectra suggesting that aggregation at that temperature is mostly unrelated to changes in secondary and tertiary structure (Fig. S2, *C* and *D*).

### Effects of drugs C and G on the aggregation of total mouse γ-crystallins (MγS) upon oxidation with H_2_O_2_ as well as the thermal aggregation of WT and F9S/Opj mutant recombinant mouse γ-crystallin

In preparation for possible *in vivo* testing, the drugs were tested against native mouse γ-crystallins. Total gamma crystallins (2.5 mg/ml) were incubated with H_2_O_2_ for 18 h at 37 °C with increasing concentrations of drugs X, C, and G, or a combination thereof. Oxidative stress–mediated aggregation at 600 nm was suppressed by drug C but poorly by drug G (Fig. S4*A*, *left panel*). Drug C in combination with G or X was most effective, but these effects were observed only above 100 μM (Fig. S4*A*, *right panel*). The baseline increase at 50 μM could reflect a destabilizing effect of the drugs because of a pro-oxidative mechanism followed by stabilizing chaperone-like effect. Systematic comparison of recombinant WT mouse S (MγS) and its cataract-prone OPJ/F9S mutant (OPJ-MγS) showed that drugs C and G were potent aggregation suppressors at or above 50 μM concentration (Fig. S4, *B*–*G*). End-point heat-mediated turbidity assay (72 °C, 20 min) shows that drugs C, G, and D have antiaggregation activity toward both WT- and OPJ-MγS (Fig. S4*B*, *left and right panels*; Table S5). There is 52.6% more aggregation of OPJ-MγS in comparison to WT-MγS under same conditions of thermal stress. Similarly, DLS showed strong suppression of radius increase by C and G for both WT-MγS and OPJ-MγS (Fig. S4*C*, Tables S6 and S7). The DLS temperature scan (30–90 °C) results (Fig. S4*D*) also corroborate previous results with HγD (Fig. 2*B*) whereby strong suppressive effects in aggregate formation were noticed with drugs C and G, mild suppressive effects were observed with drugs D, B, X, and no suppression was observed with drug M (Fig. S4*D*, *left and right panels*). Thermal denaturation study showed increase in *T*_m_ of WT-MγS by drugs in the order G > C > D > B > X > M (Fig. S4*E*, *left panel*; Table S8). Interestingly, thermal unfolding of OPJ-MγS followed a two-state unfolding pattern suggestive of N-terminal unfolding first followed by C-terminal unfolding. Drugs C and G showed a significant improvement in elevating the unfolding temperature of the C-terminal domain in comparison to the N-terminal domain (Fig. S4*E*, *right panel*; Table S8). Drug M showed no change and hence served as a negative control. Fluorescence microscopy using Nile Red provides a pictorial rendition of the type and size of aggregates formed on heating WT-MγS and OPJ-MγS, with and without drugs (Fig. S4*F*). We observed an apparent decrease in aggregates in the presence of drug C and G, whereas drug M shows little impact (Fig. S4*F*, *upper and lower panels*). The bis-ANS fluorescence inhibition pattern is again in complete agreement with the previous results for HγD (Fig. 2*F*). From the bis-ANS–binding studies of WT-MγS (Fig. S4*G*, *middle panel*) and OPJ-MγS (Fig. S4*G*, *right panel*), inhibition in bis-ANS fluorescence intensity by our selected drugs followed the order G > C > D > B > X > M, suggesting the competition between the drugs and bis-ANS for binding to the hydrophobic patches of WT-MγS and OPJ-MγS. Table 3 provides details about percent inhibition in binding of bis-ANS by our selected drugs. As for the reproducibility of these findings, see Fig. S7 and the warning note under Rigor and reproducibility section.

**Table 3:** Binding and thermodynamic parameters of HγD interaction with drugs C, G, and M at 25, 30, and 37 °C

Sample	*K*_SV_ (M^−1^) (×10^3^)			*K*_q_ (M^−1^ s^−1^) (×10^12^)			*K*_b_ (M^−1^) (×10^5)^			ΔG (kcalM^−1^)		
	25 °C	30 °C	37 °C	25 °C	30 °C	37 °C	25 °C	30 °C	37 °C	25 °C	30 °C	37 °C
C	4.215	4.197	4.107	0.739	0.736	0.72	4.7	3.07	1.57	−6.4	−6.2	−5.9
G	10.252	8.440	7.880	1.79	1.47	1.38	6.1	3.9	1.34	−7.9	−7.7	−7.2
M	0.288	0.507	0.680	0.051	0.08	0.012	0.00026	0.00069	0.00070	−1.9	−2.5	−2.6

### Probing conformational changes with far-UV and near-UV CD spectroscopy

Far- and near-UV CD spectra were recorded with and without drugs. WT-HγD crystallin at a concentration of 1 mg/ml in 10 mM sodium phosphate buffer (pH 7.2), alone without heating (control) as well as with heating at 72 °C for 20 min, was used. No visible change in the tertiary structure of WT-HγD was observed in either of the samples. This is because the *T*_m_ of the protein is around 80 °C (Fig. 2*C*), and therefore, heating HγD at 72 °C for 20 min did not result in unfolding of the protein. Given the fact that the far UV CD data also show minimal changes at 218 nm, these data combined suggest that WT-HγD crystallin aggregation and solution turbidity formation occur with minimal structural changes until the *T*_m_ of 80 °C is reached and that any aggregation suppression by the drugs may occur by a mechanism independent of unfolding inhibition.

### Drug C and G bind with medium to low affinity to human γD and mouse γS crystallins

Tryptophan fluorescence can be used for studying conformational changes resulting from protein–drug interactions (44). Fluorescence quenching of aromatic fluorophores in HγD and MγS by drugs C and G at three different temperatures (25, 30, and 37 °C) was studied in the presence of varying concentrations of drug C and G (0–250 μM). As shown in Figs. S5*A* and S6*A*, both HγD and MγS have a strong emission peak at 330 nm when excited at 295 nm. In both cases, quenching occurs with gradual addition of C and G at all three temperatures. Overall, quenching of Trp fluorescence emission is at highest at 25 °C (Figs. S5*A* and S6*A*) followed by 30 °C and lowest at 37 °C. The Stern−Volmer quenching constant (KSV) and binding constant (*K*_b_) of HγD and MγS for drugs C, G, and M at all three temperatures were obtained using the linear (Equation 1) and modified Stern−Volmer equations (Equation 2), respectively:

(1) $$\frac{F_{0}}{F}=K_{\text{sv}}\left[Q\right]+1=K_{\text{q}}\tau_{o}+1$$

(2) $$\text{log}\left(F_{\text{o}}\text{/}F-1\right)=\text{ log }K_{\text{b}}+\text{ n log }\left[Q\right]$$

where *F*_o_ and *F* are the fluorescence intensities in the absence and presence of drugs C, G, and M. *K*_SV_ is the Stern−Volmer quenching constant, and *K*_q_ is the bimolecular rate constant. *τ*_o_ refers to average integral fluorescence lifetime of tryptophan, which is ∼5.7 × 10^−9^ (45). The slope of plot *F*_o_/*F versus* [*Q*] gives *K*_sv_ (Figs. S5*B* and S6*B*), whereas *K*_b_ is obtained from intercept of plot log (*F*_o_/*F* − 1) *versus* log[Q] (Figs. S5*C* and S6*C*). As shown in Tables 3 and 4, the values of *K*_b_ for drug C and G are of the order of 10^5^ M^−1^ and decrease with increasing temperature (suggesting static binding) (46). For drug M, *K*_b_ values are of the order 10^2^ M^−1^ only. Fluorescence quenching can be either because of random collision between the molecules (dynamic interaction) or because of actual affinity between a protein pocket and drug (static interaction). Dynamic interactions are weaker and nonspecific in nature, whereas static interactions are stronger and more specific (47). For more information on the type of interaction, *K*_q_ was calculated and compared with the standard value of 2 × 10^10^ M^−1^s^−1^, which is the maximum scatter collision quenching constant of various quenchers with biopolymers (48). The *K*_q_ values for drug C, G, and M interactions with HγD and MγS as calculated from Stern–Volmer equation is greater than this value (Tables 3 and 4) suggesting static interactions. This shows that for all the drugs (C, G, and M), quenching is not initiated by dynamic diffusion but *via* formation of a complex between the drugs and the protein (HγD/MγS). Furthermore, the differential response of *K*_sv_ toward temperature change also clarifies the type of quenching. In static quenching, *K*_sv_ decreases with an increase in temperature because of the formation of complex with protein, which undergoes dissociation on increasing temperature. However, for dynamic quenching, *K*_sv_ increases with temperature as in this case higher temperature results in faster diffusion of quencher and hence larger extent of collisional quenching (49).

**Table 4:** Binding and thermodynamic parameters of MγS interaction with drugs C, G, and M at 25, 30, and 37 °C

Sample	*K*_SV_ (M^−1^) (×10^3^)			*K*_q_ (M^−1^ s^−1^) (×10^12^)			*K*_b_ (M^−1^) (×10^5)^			ΔG (kcalM^−1^)		
	25 °C	30 °C	37 °C	25 °C	30 °C	37 °C	25 °C	30 °C	37 °C	25 °C	30 °C	37 °C
C	4.828	4.249	2.988	0.847	0.745	0.524	1.38	0.327	0.0726	−7.0	−6.2	−4.1
G	7.283	8.034	4.905	1.37	1.4	0.86	1.37	0.123	0.35	−8.2	−5.6	−6.5
M	0.793	0.733	0.491	0.14	0.13	0.86	0.00093	0.00075	0.00035	−2.7	−2.6	−2.2

### Free energy change calculations show exothermic and spontaneous binding of drugs to recombinant γ crystallins of human and mouse

To gauge the strength of the major forces involved in drug–protein complex formation, change in free energy was calculated using Gibbs–Helmholtz equation (Equation 3).

(3) $$\Delta \text{G}\;=\;-\text{RT}\ln\;K_{\text{b}}$$

(50) where, *R* is gas constant and its value is 1.987 kcalM^−1^, *T* is absolute temperature (Kelvin) and *K*_b_ is binding constant whose value is already calculated using Equation 2. Negative values of ΔG for both HγD and MγS interactions with drugs C, G, and M suggest spontaneous and exothermic nature of the process (Tables 3 and 4). Free energy changes are maximum for G followed by C and M suggesting drug G to be strongest binding candidate. Free energy changes are negative at all three temperatures, being maximal at 25 °C. For C, G, and M interaction with HγD, ΔG values were −6.4, −7.9, and −1.9 kcal/mol (at 25 °C), that is, similar to those obtained by molecular docking as described later. These values also reflect the fact that drug M had the least protective effect on unfolding or aggregation of various γ-crystallins (Figs. 2 and S4).

### Molecular docking reveals two binding sites in human γD and γS protein for drugs C and G

**Figure 4:**
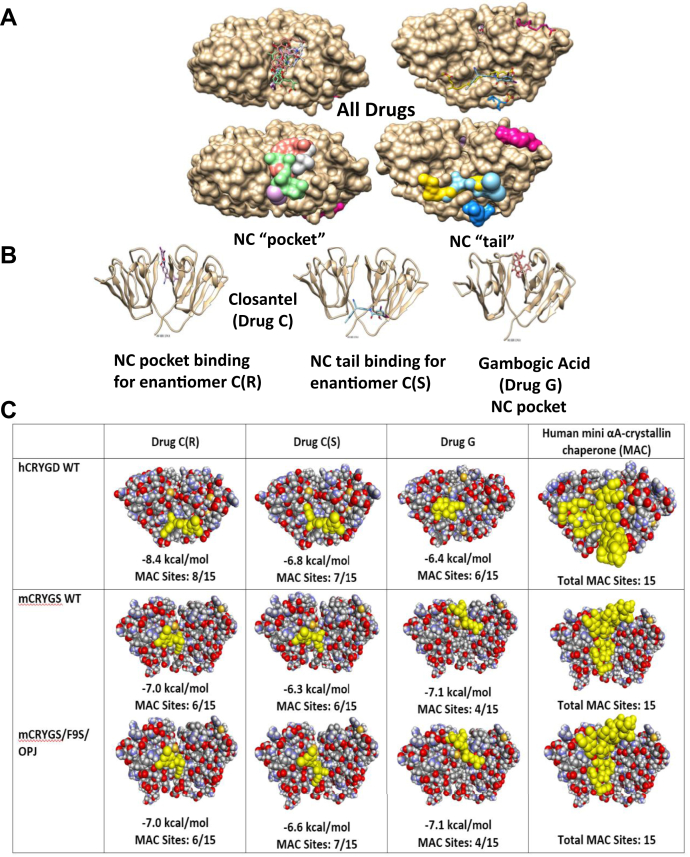
**Graphic rendition of the interactions.** Interactions between (*A*) the six candidate drugs listed in Table 1 with hγD space-fill model obtained by molecular docking. *B*, the enantiomers of drug C(S) and C(R) and drug G with the ribbon model of HγD. *C*, the binding sites of drugs C and G to HγD and MγS compared with the binding sites of the mini-αA-crystallin chaperone (MAC) peptide “KFVIFLDVKHFSPEDLTVK” obtained by molecular docking using Autodock Vina software. The N-terminal domain of the gamma crystallin models is on the *left*. *A* shows that most drugs bind either in the NC pocket or NC tail of the protein, and *B* shows that drugs C and G can bind both sites. Drug M (*magenta*) and drug T (*dark blue*) that had no protein stabilizing activity bind only to the C domain (M) or at the tip of the molecule (T), respectively. *C* shows significant overlap between the binding regions for drugs C and G onto human and mouse CRYGD and CRYGS and its mutant CRYGS/F9S. The number of shared binding sites with the 15 MAC sites are provided. Computed binding affinities are listed in kilocalorie/mole. Information on specific binding sites is provided in Tables 5 and 6. HγD, human gamma D.

Molecular docking of the drugs onto HγD protein structure surprisingly revealed two major binding sites, one in the pocket between the N and C domain (NC-P) where five of the seven drugs (C, G, D, H, and B) were typically binding to residues spanning the region 50 to 158, and one we named the NC-tail spanning domain (NC-T) typically spanning 56 to 61, 107 to 109, and 168 to 174 in which same compounds also bind (Fig. 4, *A* and *B*). Thus, each compound often had both a higher-affinity and a lower-affinity binding site. Note that drug M binds to sites unrelated to NC-P or NC-T.

In order to understand the relevance of these sites for the chaperone activity of drugs C and G, we docked the sequence of the mini-αA-crystallin chaperone (MAC) peptide ^70^KFVIFLDVKHFSPEDLTVK^88^ (MAC) (51) onto the HγD, MγS, and the F9S mutant (OPJ-MγS) crystal structure (Fig. 4*C*). This sequence has been shown to have chaperone activity and to protect γ-crystallins against thermal- and oxidation-mediated aggregation (52, 53). This figure conveys a visual rendition of where the drugs bind in relationship to the MAC binding sites. The specific amino acid residues in CRYDG are displayed in Tables 5 and 6. Focusing on drugs C and G, the enantiomers of closantel C(S) and C(R) each have binding sites to both domains of HγD, with C(R) having higher affinity (−8.4 kcal/mol) than C(S) for the NC-T compared with the NC-P domain (−6.8 kcal/mol). Drug G shares up to 11 binding sites with drug C in the NC-P domain, each spanning residues 50 to 158 (*i.e.*, 50, 51, 54, 79, 145, 147,150, 151, 156, 157, and 158), but it also has binding affinity to the NC-T domain, where it shares five residues (58, 168, 172, 173, and 174) with closantel C(R) but with lower affinity (−6.4 kcal/mol) than to the NC-P (−7.2 kcal/mol). Note that hematoporphyrin (H) had similar NC-P binding sites as gambogic acid (G), but we chose not to study it further because of its strong red color and known deleterious photosensitizing properties (54) and thus potential cataractogenic agent. In contrast, drug M bound neither to the NC-P domain nor the NC-T domain, likely explaining thereby why it had poor stabilizing properties toward stressed γ-crystallins throughout the aforementioned studies.

**Table 5:** Binding affinities of lead compounds (score as kilocalorie/mole) and residue interactions to HγD NC-pocket domain

NC-pocket binding sites (no MAC binding sites)																							
Drug	Score	P 49	N 50	Y 51	S 52	G 53	Q 55	R 80	R 142	Y 144	L 145	L 146	M 147	P 148	D 150	Y 151	R 152	Q 155	D 156	W 157	G 158	A 159	T 160
B	−6.3																				**X**		
C(S)	−7.8		**X**	**X**	**X**	**X**	**X**	**X**	**X**	**X**	**X**		**X**		**X**	**X**			**X**	**X**	**X**		
C(R)	−7.8		**X**	**X**	**X**	**X**	**X**		**X**	**X**	**X**	**X**	**X**		**X**	**X**			**X**	**X**	**X**		
D	−7.0		**X**	**X**	**X**		**X**	**X**					**X**		**X**		**X**		**X**		**X**	**X**	**X**
G	−7.2	**X**	**X**	**X**			**X**	**X**			**X**	**X**	**X**	**X**	**X**	**X**		**X**	**X**	**X**	**X**		
H	−7.6		**X**	**X**	**X**	**X**	**X**	**X**		**X**	**X**		**X**		**X**	**X**	**X**			**X**	**X**		
Bis-ANS	−7.8		**X**	**X**	**X**	**X**	**54**	**79**	**X**	**X**			**X**			**X**			**X**	**X**	**X**		

For comparison, MAC binding sites uncovered by molecular docking are indicated in bold.

**Table 6:** Binding affinities of lead compounds (score as kcalorie/mole) and residue interactions to HγD NC-tail spanning domain

NC-tail binding sites (12 MAC binding sites indicated in bold)																													
Drug	Score	Y 56	**L** **58**	**R** **59**	R 60	**G** **61**	**W** **69**	**S** **85**	**E** **107**	**D** **108**	C 109	S 110	Q 113	R 117	N 119	E 128	E 135	L 136	S 137	Y 139	R 163	V 164	G 165	**R** **168**	**R** **169**	**I** **171**	**D** **172**	**F** **173**	S 174
B	−6.3	**X**	**X**	**X**	**X**	**X**				**X**	**X**					**X**				**X**				**X**		**X**		**X**	**X**
C(S)	−6.8	**X**	**X**						**X**	**X**		**X**								**X**				**X**	**X**	**X**		**X**	
C(R)	−8.4	**X**	**X**						**X**	**X**										**X**				**X**	**X**	**X**	**X**	**X**	**X**
G	−6.4		**X**	**X**	**X**		**X**																	**X**			**X**	**X**	**X**
H	−7.6																							**X**					
M	−4.0												**X**	**X**	**X**		**X**	**X**	**X**		**X**	**X**	**X**						
X	−5.8							**X**																	**X**	**X**	**X**	**X**	
Bis-ANS	−7.1	**X**	**X**						**X**	**X**	**X**	**X**								**X**				**X**	**X**	**X**	**X**	**X**	

Note: Drug X has additional binding sites at residues P83, H84, G86, S87, H88, and R89. Residues that are shared with MAC binding sites are in bold.

Extensive studies were also carried out with MγS and its mutant F9S, whereby only 15 MAC sites were present in both the mouse WT-MγS and its cataract-prone F9S/OPJ mutant. Drugs C(R), C(S), and G had only six, six, and four binding sites for MγS and six, seven, and four sites, respectively, for the F9S/OPJ mutant, respectively. Thus, one would expect these drugs to be potentially less effective against mouse *versus* human γS.

Attempts to verify the binding sites of drug C onto HyD by NMR failed. For this reason, a trypsin digestion experiment using 400 μM drug G was carried out in the hope to identify miscleavage sites. Drug G rather than C was chosen for these experiments since it best inhibited turbidity formation when, in a separate experiment, the samples were exposed to thermal stress at 72 °C for 20 min. The samples incubated with or without 400 μM drug G were digested for 4 h with sequencing grade trypsin, and the tryptic peptides were analyzed using LC/MS/MS since no specific fragment was noted using SDS-PAGE. As shown in Table S9, miscleavage sites were observed at R60, R80, R140 and in the telopeptide region 154 to 174 compared with no drug control, though the reliability of the 154 to 174 assignment is limited. Though good matches of full mass spectra were observed, MS/MS assignments are limited probably because of the multiple miscleavage of Arg residues in this peptide and therefore the high charge states of the precursor ions of 603.5609 (4+). Both R80 and R168 are drug G docking sites providing thereby a partial answer to the question of binding sites.

### Inhibition of aggregation of oxidized HγD by bis-ANS and drugs C, G, and M

In postsubmission experiments, we attempted to find mechanistic clues relevant to the use of an H_2_O_2_ oxidation step prior to the thermal stress used during the initial drug screen. For this reason, we did a comparative study of oxidized (HγD-ox) *versus* native HγD (HyD-nat), hypothesizing that bis-ANS would both act like the drugs and provide information on the role of exposed hydrophobicity (40). HγD was oxidized in diethylenetriaminepentaacetic acid (DTPA)-treated phosphate buffer to facilitate hydroxyl radical formation and protein damage, followed by dialysis against DTPA-treated buffer to eliminate the known proaggregating effects of divalent metals (32) in the subsequent steps. SDS-PAGE indeed detected small increases in MW both in the absence and presence of reducing agent in HγD-ox, but not HγD-nat, suggestive of cleavage of internal disulfides (Fig. 3*A*). While proteomic sequencing analyses are pending, Figure 3, *E* and *F* shows that 100 and 500 μM bis-ANS potently inhibited aggregation of HγD-ox incubated for 10 min at 72 °C, better than HγD-nat and 100 μM drug C. Overall protective effects were more pronounced for the oxidized than nonoxidized form of HγD. Unfolding studies using 360/320 nm ratio in the presence of 100 μM agents (Fig. 3*B*) revealed that HγD-ox unfolded with *T*_m_ of 68 ± 0.07 °C, which increased to *T*_m_ of 78 ± 0.1 °C with bis-ANS, whereas HγD-nat unfolded at *T*_m_ of 78 ± 0.1 °C, which increased to 82 ± 0.02 °C. Most powerful were drugs C and G (but not M), which increased all *T*_m_ values to the maximum value of both protein forms. Exposure of hydrophobic sequences probed with bis-ANS (Fig. 3*C*) revealed little effect of heating on the HγD-nat until 95 °C was reached but massive increase of bis-ANS fluorescence starting already at 35 °C in HγD-ox. A graph comparing unfolding based on 360/320 nm fluorescence ratio *versus* bis-ANS fluorescence (Fig. 3*D*) illustrates dramatic differences between HγD-ox and HγD-nat suggestive of substantial exposure of hydrophobic sequences long before *T*_m_ is reached. In the hope to obtain clues on the impact of bis-ANS, drugs C, G, and M on protein aggregation status, native PAGE was performed (Fig. 3*G*). Polymers greater than 250 kD are present in unheated HγD-nat but only the size of 75 to 100 kD in HγD-ox. By and large upon heating, all staining disappeared in all samples (except drug G-ox), suggesting that Coomassie binding sites were masked by the thermal process. However, a faint band is visible at 50 to 75 kDa in the presence of bis-ANS suggesting partial suppression of aggregate size. Other techniques will be needed to assess the relationship between drugs and aggregate size.

## Discussion

This study is to our knowledge the first to screen Federal Drug Agency approved and experimental drugs for the existence of small molecules with α-crystallin-like chaperone activity toward γ-crystallins as a paradigm for potential pharmacological inhibition of cataract. However, it should be pointed out that a similar screen was used by Mackley *et al*. (55) to search for drugs that improve α-crystallin chaperone activity. Whereas all six compounds obtained by library screening had some activity toward total bovine γ-crystallins in the initial screen, closantel (drug C) and gambogic acid (drug G) emerged as the most robust and active agents with broad antiaggregation activity toward both recombinant γD and γS crystallins from human and mouse as well as their respective mutants (W43R, R14C, R58H of HγD and F9S/OPJ of MγS), except for W43R toward drug C. Closantel (C) is an antihelminthic drug approved for veterinary use, whereas gambogic acid (G) is a xanthonoid derived from the brownish-orange resin extracted from the bark of *Garcinia hanburyi*, a tree growing in South East Asia https://en.wikipedia.org/wiki/Gambogic_acid. Both have a similar MW, that is, 685 Da and 628 Da, respectively, and a complex system of aromatic rings with functional groups that engage several amino acid residues shared with the MAC sequences docked onto HγD and HγS. Gambogic acid and similar xanthones from Garcinia bark extract have been found to inhibit the aggregation of various proteins (56, 57). Of interest is that our screen was also positive for hemin (protoporphyrin IX with Fe^3+^) and its derivative hematoporphyrin. Hemin was previously identified as a generic and potent broad protein misfolding inhibitor with activity toward amyloid fibril formation and amorphous aggregate formation by alcohol dehydrogenase, catalase, and gamma crystallin (58). We did not further study the compound because of its known photosensitizing properties that would have made it unsuitable for application to the eye.

Several studies by others have reported the existence of small molecules that bind to γ-crystallins, including lanosterol (59), cochineal carmine (60), ortho-Vanilin (61), quercetin (62), hesperetin (63), and sodium citrate (64). Moreover, small molecules with either demonstrated potential anticataract properties involving antiaggregation activity against crystallins include rosemarinic acid (65), morin (66, 67), 25-hydrocholesterol (68), epigallocatechin gallate (69), and myoinositol (70). Among these compounds, those that were included in the screen included epigallocatechin gallate, which improved aggregation, whereas morin, lanosterol, hesperetin, and carmine had no impact. Surprisingly, quercetin actually worsened aggregation (Table S10).

One question of great importance is that of the mechanism by which our drugs inhibit the aggregation of the gamma crystallins described in this study. At first, it should be pointed out that we have loosely used the term aggregation to explain turbidity, but the latter can result from processes as varied as isoelectric precipitation, phase separation, flocculation, denaturation, and crystallization (71), whereby the molecular processes underlying these may or may not be due to irreversible modifications of the protein itself. Moreover, the structural heterogeneity of the top six drugs described above is such that a unifying mechanism of protection is unlikely to emerge. Yet, in-depth studies on oxidation, photo-oxidation, low pH, divalent metals, and heat-induced mechanisms of aggregation and chaperone protection of HγD and its cataract-prone variants by the groups of King, Shakhanovich, Pande, Sharma, and others, all cited in this work, now offer a molecular framework for a tentative understanding of how our drugs might protect stressed or mutated gamma crystallins.

From a chaperone perspective, both C and G have binding affinities to MAC binding sites, as revealed by molecular docking. Yet, some caution is needed since the docking sites of C and G only partly agree with the MAC sites mapped by NMR spectroscopy by Banerjee *et al.* (53). The latter are Y6, D38, Q54, F56, D61, D97, E107, S123, S130, V132, N138, R152, V164, L167, S174, whereas we found Q54, F56, E107, and S174 being shared with drug C, but only Q54 and S174 being shared with drug G. At high temperature, these authors found that binding between MAC and HγD was irreversible and involved F56, V132, V164, G165, S166, and L167. However, attempts to confirm drug C binding sites by NMR spectroscopy using ^15^N-labeled HγD in 5% DMSO failed. This could be, in part, because of the differences in the NMR experiment conditions *versus* those of the molecular docking simulation, whereby temperature and solvent (water and DMSO) were not identical. More likely, however, γ-crystallins have robust stability that is unlikely to change upon binding to small molecules of low to medium binding affinities. The ability of γ-crystallins to resist changes in their structure was demonstrated by the ^1^H,^15^N heteronuclear single quantum coherence (HSQC) spectrum in studies of WT *versus* their deamidated mutants as studied by Guseman *et al.* (72). Moreover, there are to our knowledge no comparative data in the literature on drug binding sites to γ-crystallins by NMR spectroscopy. While NMR studies failed to identify specific cleavage sites for drug C, several of the trypsin miscleavage sites closely match gambogic acid (drug G) binding sites uncovered by molecular docking using Autodock Vina (Scripps Research; https://vina.scripps.edu/LICENSE/), that is, R60, R80, and R168 (Tables 5 and 6). Thus, this experiment provides a partial answer to the issue of experimental identification of specific drug binding sites on HγD crystallin.

From the fluorescence microscopy data, it would appear that drugs C and G are able to partly prevent initial unfolding of the C-terminal domain at elevated temperature. Below 72 °C based on the DLS, it appears that the drugs partly prevent the transition from small to large MW aggregates, whereby no major conformational changes are observed based on the CD spectra of HγD. A detailed mechanism of high MW aggregate formation has been described by Watzky and Finke (73). The bis-ANS binding studies suggest that the drugs have affinity for the hydrophobic residues in the NC pocket/tail in both HγD and MγS. Thus, it may be possible that our drugs bind to the hydrophobic residues of small MW aggregates, thereby preventing their combining with each other through hydrophobic interactions, which eventually results in amorphous high MW aggregates. While, it is not possible to tell whether binding at the NC-P or NC-T sites is more important for protection of γ-crystallins, binding to the NC-T spanning domain, residues R59 (perhaps Q54) to 169, 171, 172, and S174 (Tables 5 and 6) appear to be particularly attractive targets for future drug development. However, as discussed later, some of these binding sites may have to be revisited.

**Figure 5:**
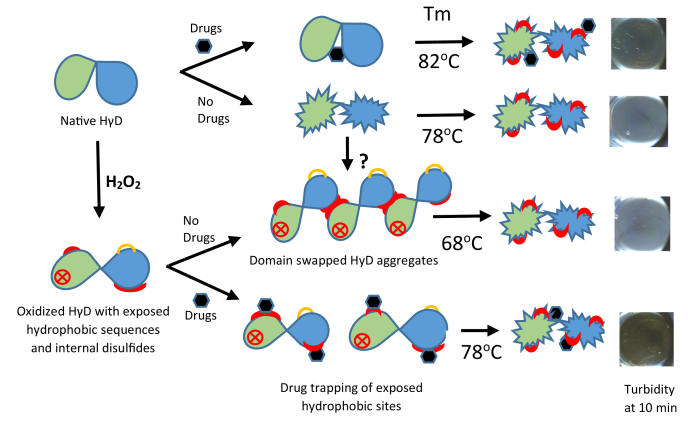
**Tentative mechanism by which the α-crystallin mimetic drugs might prevent solution turbidity of oxidized and native HγD using bis-ANS as a proxy for the drugs based on our findings that it potently suppresses heat-induced turbidity.** A mechanism based on domain swapping (74) and exposure of hydrophobic bis-ANS binding sequences is proposed. In the oxidized protein, the latter were mostly exposed already below the *T*_m_ of HγD-ox, whereas no such exposure was noted for HγD-nat until the *T*_m_ was reached. Yet in both cases, bis-ANS, like drugs G and C, was able to increase the *T*_m_ of both oxidized and native HγD. Based on Serebryany’s work, internal disulfides are likely present in HγD-ox (74). However, aggregates seen by native PAGE (Fig. 3*G*) are unlikely crosslinked by external disulfides since these were absent from HγD-ox by SDS-PAGE. Extensive studies will be needed to probe the aforementioned tentative mechanisms and role of aggregates in light of current knowledge of factors that impact on the stability of HγD N- and C-terminal domains. Legend: *red circle with cross*: generic oxidative damage; *red color*: postulated exposed hydrophobic domains; *orange half-circles*: internal disulfides; and *black six-membered ring*: drug. Note that the images showing the presence or absence of protein aggregates at the bottom of the wells are identical to those of Figure 3*E*. They are being reused here for the sake of linking the proposed aggregation mechanism with a real instead of a simulated picture. bis-ANS, bis-8-anilino-1-naphthalene sulfonic acid; HγD, human gamma D.

Additional mechanistic information comes from our studies with bis-ANS as potential drug proxy for inhibition of turbidity in solution of oxidized HγD. Based on the Serebryany studies using oxidation mimetic HγD mutant W42Q and W42R, it would appear that oxidative damage would favor aggregation-prone structures *via* domain swapping and formation of deleterious C32–C41 internal disulfide (34, 74). Based on this work, in Figure 5, we tentatively propose that the damaged HγD would undergo partial unfolding of the N-terminal domain favoring binding to hydrophobic C-terminal domain and that drugs would prevent growth of the aggregates by trapping the hydrophobic sites, thus aborting the growth of the domain-swapped aggregates. In partial support, there is massive binding to HγD-ox at temperatures below the *T*_m_ (Fig. 3*D*) and a faint hint of lower size aggregates in the bis-ANS lane of the native gel (Fig. 3*G*). In contrast, native HγD unfolded only at the *T*_m_ of 82 °C most likely by a mechanism unrelated to oxidation. Here again, we propose that the drugs stabilize the domain interface or possibly trap C-terminal hydrophobic sequences as soon as they are exposed.

These propositions are tentative at this time, and the question of the extent to which bis-ANS is a proxy for our drugs will need to be investigated in light of the dissenting data from Banerjee *et al.* (40) who determined its NMR binding sites to HγD and the cataract-prone V76D mutant. These authors showed that bis-ANS binds native HγD residues in an N-terminal cluster (G10, F56, R58, R59, A63, Q67, M69, and G70) and a C-terminal cluster (H88, R117, F118, N119, Y134, E135, G141, R142, and D150), which considerably differ from those identified by Autodock Vina (Tables 5 and 6). Thus clearly, advanced studies will be needed to fully unravel the mechanisms of aggregation inhibition. One reason why such demonstration might be quite challenging and time consuming to make is the unexpected finding that agaric acid (drug A), a compound with detergent properties and low binding affinity score to HγD (−5.1 kcal/mol for NC pocket and −4.7 kcal/mol for NC tail) actually protects BGC at 10 min and both HγD and MγS at 20 min heating (Fig. S7). Similarly, drug M, which we used mostly as a negative control with no activity on *T*_m_, actually protected oxidized HγD (Fig. 3*E*). Thus, these detergent-like aliphatic chain compounds might have protective effects on turbidity by different mechanisms, perhaps *via* micelle formation.

One limitation of this study that is common to all small-molecule screening strategies is that the target of the candidate drugs is potentially very narrow and therefore of limited use for the prevention of cataract resulting from multiple insults and mechanisms of aggregation. It is actually possible that we may have identified molecules that protect the human but not the mouse lens since we found less drug-binding sites in mouse (*i.e.*, 6 and 4 for drug C and G, respectively) *versus* human γS-crystallins (13 and 11 for drug C and G, respectively). The promising outcome is that drugs C and G have relatively broad beneficial effects on both γD and γS from either human or mouse origins, perhaps because we screened against total bovine crystallins rather than a single recombinant species. However, the low binding affinities mean that it will be difficult to achieve high *in vivo* concentrations in order to block unfolding and aggregation. This may be a problem for many of the crystallin mutants that unfold at lower temperature, such as W43R in which drug C had deleterious effects. Moreover, the requirement of high DMSO concentrations for drug solubility may result in toxicity. Successful studies with mice models prone to cataract formation will hopefully support the initial premise of this work.

Another limitation is that the concept aggregation inhibitors as treatment paradigm for cataract faces biological and pharmacological challenges in view of the work of Schmid *et al*. (75) on the one hand, and Truscott *et al.* (76) on the other hand. Schmid *et al.* reported a relative rapid loss of γ-crystallins because of proteolytic activity in the initial months of life of cataract-prone mice with mutant gamma crystallin, such as γD-V76D. They propose that opacification of the lens is the result of an imbalance in the overall composition of lens crystallins rather than a process resulting from aggregation of the mutant protein. If correct, this would suggest that inhibitors of congenital cataract should seek to inhibit proteolysis rather than aggregation of the mutant protein. Yet, while many studies using antioxidants, cholesterol derivatives, and other experimental drugs are successful at preventing cataract progression in the rodent lens, the human lens develops with age a diffusion barrier such that the movement of cysteine, glutathione, and drugs from the cortex to the nucleus is severely hampered (76, 77). This diffusion barrier is likely beneficial to prevent low-MW toxins from reaching the nucleus but constitutes an impediment toward the pharmacological inhibition of age-related and congenital cataract.

In summary, we believe that this work provides a basis for the existence of drugs and small molecules with α-crystallin chaperone activity toward gamma crystallins that is compatible with chaperone mimetic behavior. However, the extent to which this mimicry is identical at the molecular level remains to be established. Similarly, their promise as inhibitors of congenital and age-related cataract formation remains to be proven.

## Experimental procedures

### Chemicals

Sephadex G-200 (discontinued product) was originally obtained from Pharmacia Biotech (now GE Healthcare). The Microsource Spectrum Collection and small-molecule library containing 2560 compounds included in the US and International Drug Collections, together with Natural Product and Discover libraries, was purchased from Microsource Discovery Systems, Inc. DMSO and H_2_O_2_ (30%) were from Thermo Fisher Scientific. DTPA and all other chemicals were from Sigma–Aldrich and were from the highest available purity grade. Specialty chemicals (>95% purity guarantee by manufacturer) present in the Microsource library that were purchased in powder form included the following: gambogic acid (drug G) from Shanghai Tauto Biotech Co, closantel (drug C), hematoporphyrin (drug H), hexachlorophene (drug X), and docusate sodium (drug DS) from MCE (MedChemExpress), dihydrogambogic acid (drug D), chaulmoogric acid (drug M) and hematein (drug I) from MicroSource, Inc, bixin (drug B), sennoside A (drug S), tetradecyl sulfate sodium (drug T), agaric acid (drug A) and escin (drug E) from Sigma, avocadene (drug V) and all other drugs were taken directly from the Microsource microtiter plate library as 10 mM solutions in DMSO.

### Isolation and purification of total gamma crystallins from bovine and mouse lens

Calf lenses (∼3 years old) were obtained from a local abattoir, freed from connective tissue, and stored at −80 °C. Eyes were thawed in warm water, and lenses were processed according to Ortwerth *et al*. (78) with modifications. Lens cortex and nucleus (bovine only) were physically separated and homogenized in 30 ml cold water using a hand-held glass homogenizer under a stream of argon. The homogenate was centrifuged for 30 min at 30,000 rpm at 4 °C, and supernatant and pellet were separated. The supernatant was dialyzed at 4 °C overnight against 4 l of 50 mM K_3_PO_4_ buffer at pH 7.2 (buffer A) prior to loading onto a Sephadex G200 column. Total mouse lens γ-crystallins were similarly isolated and purified from a pool of 200 lens from C57BL6 adult mice at autopsy as approved by Institutional Animal Care and Use Committee protocol 042-2015 to V.M.M.

### Gel filtration chromatography

A Sephadex G-200 column was packed into a 5 cm × 60 cm BioRad glass column (Bio-Rad) equilibrated at 4 °C in buffer A with a flow rate of 40 ml per min whereby 10 to 15 ml fractions were collected using a Pharmacia Model 700 pump (Pharmacia/GE Healthcare) according to Senthilkumar *et al.* (79). Lens homogenate (25–30 ml) was chromatographed, and protein concentration was manually measured using a Nanodrop 2000 spectrophotometer (Thermo Fisher Scientific). A typical profile of cortical and nuclear crystallin is shown in Fig. S1*A*. Fractions were collected, pooled, and concentrated using Amicon centrifugation filters with a 10,000 Da MW cutoff membrane. This one-step procedure generally achieved 95% purity as judged by SDS-PAGE, whereby further purification was sometimes needed by rechromatography over the G200 column or by cation exchange chromatography using Toyopearl GigaCap S-650M column (TOSOH) as previously described (80).

### Expression and purification of human γD-crystallin and mice γS-crystallin and their mutants

HγD in pET3d vector was gifted from Dr Noriko Fujii†. MγS and its mutant OPJ-MγS in pET17b vector was obtained from Dr Graeme Wistow. Expression and protein purification was carried out as described previously with slight modifications (81). WT and mutant proteins were transformed into BL21(DE3) pLysS cells (Thermo Fisher Scientific). A recombinant protein synthesis was induced with 0.5 mM IPTG. Induction was done for 5 h at room temperature followed by harvesting of cells (centrifugation at 7500 rpm for 10 min). Pellets obtained were washed with Tris buffer (pH 8) and stored at −80 °C until use. Cells were lysed by a sonication cycle of 30 s pulse and 30 s rest for 20 min in 20 mM Tris–HCl, pH 8 containing 1 mM EDTA, and 1 mM PMSF. Lysate was centrifuged at 12,500 rpm for 40 min. Supernatant was collected and filtered with 0.45 μm and 0.22 μm syringe filters. The filtered lysate was purified using gel filtration over Sephadex G-200 followed by cation exchange chromatography using Toyopearl GigaCap S-650M column (TOSOH).

### Site-directed mutagenesis

The HγD crystallin mutants used in this study (R14C, R58H, and W43R) were generated by site-directed mutagenesis using the Mut Express II Fast Mutagenesis Kit V2 (Vazyme) following the manufacturer’s protocol. The primer pairs used for mutagenesis are listed in Table S4. All mutagenesis sites were validated by DNA sequencing analysis, SDS-PAGE (Fig. S2*A*), and Western blot (Fig. S2*B*).

### SDS-PAGE

Protein purity was assessed using SDS-PAGE and a 14% gel with a NOVEX electrophoresis apparatus (Thermo Fisher Scientific). HγD crystallin and its various mutants typically migrated as 17.5 kDa band (Fig. S2, *A* and *B*). WT-MγS and OPJ-MγS migrated as 20 KDa bands (Fig. S3, *A* and *B*). The same electrophoresis system but using the manufacturer’s native gel loading sample and running buffers without SDS was used to perform native protein electrophoresis of HγD and its oxidized form upon exposure to thermal stress in the presence of drugs or bis-ANS. Both SDS and native gels were developed with Coomassie stain as previously described (80)

### Western blotting

HγD, MγS, and their mutants separated by 14% SDS-PAGE gels and proteins were transferred to nitrocellulose membrane (EMD Millipore) at 100 V for 100 min. The membrane was blocked with 5% (w/v) skimmed milk in PBS with 0.1% Tween-20 (PBS-T). The membrane was then incubated with polyclonal anti-γD-crystallin antibody (1:5000 dilution) (Sigma) in PBS-T containing 0.1% bovine serum albumin overnight at 4 °C. After washing, the membrane was incubated for 1 h at room temperature with anti-rabbit horseradish peroxidase–conjugated secondary antibody (1:5000 dilution) (Sigma) in 5% (w/v) skim milk PBS-T. Blots were washed and detected with chemiluminescent substrate according to the manufacturer’s instructions (Thermo Fisher Scientific). Figs. S2*B* and S3*B* show the immunoblots for HγD and HγD mutants, and MγS and MγS/OPJ mutant, respectively.

### Protein assay

Protein concentration was determined *via* a NanoDrop 2000 spectrophotometer at 280 nm using the default settings corresponding to the extinction coefficient of bovine serum albumin (43,824 M^−1^ cm^−1^) that is similar with that of HgD-WT, R14C, and R58C, (42,850 M^−1^ cm^−1^), 9% lower for MGS and OPJ (40,255 M^−1^ cm^−1^, calculated), and 15% lower for W43R (37,360 M^−1^ cm^−1^). Thus, protein concentrations for these mutants have been adjusted accordingly. Other assays have used the Bicinchoninic Acid Assay kit (Thermo Fisher Scientific) using absorbance at 565 nm.

### High-throughput screen of small molecules for resistance to H_2_O_2_ and heat shock–mediated aggregation of bovine gamma crystallin-rich protein fraction

All assays were carried out manually in CORNING #9017 Costar Assay 96-well plates. Wells were filled with 90 μl calf γ-crystallin-rich protein solution (pool 4 at 2 mg/ml) followed by 0.5 to 5.0 μl of drug (all 10 mM stock solution in DMSO) as supplied by the manufacturer and 10 μl H_2_O_2_ (450 mM stock solution in water) and sealed with sticky Applied Biosystems MicroAmp Clear Adhesive films (Life Technologies). Control wells received DMSO only. Plates were shaken for 2 min and incubated from 24 to 72 h in a culture room at 37 °C. Aggregation/insolubilization was assessed by absorbance reading at 600 nm (instead of 400 nm to prevent interference by UV–visible active drugs with absorption maxima in that region) using a Tecan 2000 microtiter plate reader (Tecan). A second reading was performed following a heat shock of 20 min at 72 °C as described by Chen *et al.* (36) in order to capture drugs with heat-resistant chaperone activity. In order to quantitate the impact of the drugs on aggregation, a fitness plot was created (Fig. 1 *C*) that allowed us to identify wells with enhanced/worsening *versus* suppressed crystallin aggregation. In addition, aggregation in each well was also visually assessed by two independent “blinded” readers for the ability to read fine print through each well by placing the plates over small font text (Fig. 1*D*). Both machine and visual readouts were reconciled in order to increase robustness. To ensure reproducibility, experiments were repeated multiple times with bovine and human recombinant gamma crystallin preparations at lower concentrations whereby bovine α-crystallin (pool 1) was used as positive control for total suppression of aggregation. A ranking was established that resulted in identification of seven antiaggregation candidate compounds that were chosen for further investigation.

### Biophysical characterization of the effects of candidate drugs on native and recombinant γ-crystallins

#### End-point and kinetic turbidity assays

For end-point assays, HγD and MγS (1 mg/ml, 100 μl) in 50 mM K_3_PO_4_ buffer (pH 7.2) in set of triplicates in transparent flat bottom 96-well plates were heated at 72 °C for 20 min in the presence and absence of selected drugs at varying concentrations. Absorbance was measured at 600 nm with a SpectraMax iD5 (Molecular Devices).

Aggregation kinetics assays at 600 nm in the presence of varying concentrations (0, 100, 200, 300, 400, and 500 μM) of our selected drugs were also performed on mutants of HγD (W43R, W42Q, R14C, and R58H) and MγS (F9S) at a concentration of 1 mg/ml in 50 mM K_3_PO_4_ buffer (pH 7.2) in a total volume of 100 μl. WT-HγD and WT-MγS crystallin served as controls for their respective mutant versions. For W43R mutant, aggregation assay was done at 42 °C for a total of 200 min. Absorbance was monitored every 10 min. For R14C and R58H mutants, end-point turbidity assay was done at 72 °C for 20 min. Both the mutants were pretty stable at temperature as high as 55 to 60 °C, and no turbidity was observed even after 3 to 4 h, and hence because of the unavailability of a device that can do kinetic aggregation study at higher temperatures (65 °C and beyond), we preferred to choose end-point turbidity assays at 72 °C for 20 min. All the experiments were run in duplicates or triplicates.

### DLS

Efficacy of our selected drugs in preventing the formation of protein aggregates was measured by DLS. Measurements were performed on a Wyatt DynaPro Nanostar. For DLS temperature scan experiment, pure and filtered protein samples (1 mg/ml in 10 mM sodium phosphate buffer, pH 7.2) with and without drugs (400 μM) were analyzed by DLS over a temperature range of 30 to 90 °C. Multiple scans were run for every data point, and the results represent average of individual scans. DLS spectra were also recorded for the samples at the end point of turbidity assay.

### CD spectroscopy (secondary structure analysis)

Far-UV and near-UV CD spectra of HγD-crystallin was recorded using CD spectrometer model 215 (AVIV instruments, Inc). HγD crystallin samples (10 μM) in the presence and absence of drugs were prepared using 10 mM sodium phosphate buffer (pH 7.2). Spectra were recorded at room temperature between 190 and 260 nm using a cylindrical quartz cuvette of 1 mm path length. The represented spectra are the average of three scans, and respective blanks were subtracted from each spectra.

### Thermal unfolding

Thermal denaturation study was monitored using fluorescence spectroscopy. Protein solutions (10 μM) in the presence and absence of drugs or bis-ANS (100 μM) were heated continuously from 30 to 95 °C, and the fluorescence data were collected every 5 °C after 1 min equilibration at the given temperature. Unfolding data were fit to Greene and Pace’s two-state model (82) using GraphPad Prism software (GraphPad Software, Inc).

### Bis-ANS binding studies

#### Extrinsic fluorescence spectra of proteins

Extrinsic fluorescence spectra were recorded using bis-ANS, a well-known surface hydrophobicity probe. The spectra were recorded between 400 and 600 nm after exciting at 390 nm. The experiment was performed on 10 μM of HγD and MγS in 50 mM K_3_PO_4_ buffer with and without our candidate drugs (100 μM). Bis-ANS stock solution was prepared in methanol whose final percentage was maintained below 7 after mixing with proteins (81).

### Drug-binding studies (steady-state fluorescence quenching studies)

All the steady-state fluorescence measurements were recorded on Fluoromax Spectrofluorometer (Horiba Scientific). For fluorescence quenching measurements, 10 μM of both HγD and MγS crystallins were titrated with varying concentrations of drugs C, G, and M (0–250 μM) at 25, 30, and 37 °C. These samples were excited at 295 nm, and the fluorescence emission was collected from 310 to 410 nm.

### Fluorescence microscopy using Nile Red

Protein samples at the end point of turbidity assay were used for fluorescence microscopy. Staining was achieved with the addition of 0.5 μl of the Nile Red to 50 μl of the protein solution. Immediately after Nile Red addition, aliquots of the protein samples containing the dye were placed on Kova Glasstic slides (Hycor) and observed by confocal microscopy (38). Three-dimensional imaging was performed using a Leica SP5 inverted confocal microscope with a 20× objective (numerical aperture = 0.7), HeNe laser excitation wavelength of 543 nm, and detection wavelength of 570 to 705 nm. Images were analyzed using Imaris software from Bitplane, Inc.

### Transmission electron microscopy

Protein aggregates from the end point of turbidity assays were diluted fivefold with water. Formvar/carbon-coated EM nickel grids (400 mesh; Ted Pella) were incubated onto glow discharge for 1 min. The grids were placed on formvar/carbon side down on top of a drop of the sample solution for 1 min. The grids were removed, blotted with filter paper, and placed onto a drop of 1.0% uranyl acetate solution for 1 min. The excess uranyl acetate was removed, and the EM grids were air dried. The grids were observed by an FEI Tecnai Spirit (T12) electron microscope, and the images were captured by a Gatan US4000 4kx4k CCD camera.

### NMR investigation of potential binding sites of drug C to ^15^N-labeled HyD

HSQC experiments performed with a Bruker 800 MHz instrument to monitor ^15^N–^1^H amide bonds were conducted with 100 μM protein and 600 μM drug C in 5% DMSO over a range of temperature, reasoning that if the *K*_*d*_ is in the midmicromolar range, this should be able to detect it. HSQC between the drug-treated sample and control sample in DMSO showed no significant differences in spectra overlay, and therefore, full results are not presented.

### Identification of trypsin miscleavage sites from binding of drug G to heat-denatured HγD

HγD protein (2.5 mg/ml buffer A) was incubated at 72 °C for 20 min without or with 100 or 400 μM drug G or C in buffer A. Final DMSO concentration was 5% in all samples. At 400 μM, drug G but not C markedly suppressed turbidity (not shown). Therefore, samples with and without 400 μM drug G were chosen for identification of drug G-induced miscleavage sites upon digestion with 25:1 protein:trypsin ratio (sequencing grade modified trypsin; Promega) in buffer A. After 4 h, samples were frozen at −80 °C until processed for determination of miscleavage sites using proteomics analysis. LC–MS/MS analysis was performed on an Orbitrap Eclipse mass spectrometer (Thermo Fisher Scientific) coupled with a Waters nanoAcquity UPLC system (Waters) in total 90 min run using a linear gradient range from 0 to 42% solvent B (0.1% formic acid in acetonitrile) *versus* solvent A (0.1% formic acid in water) at a flow rate of 300 nl/min. MS1 full mass spectra were acquired in the Orbitrap mass analyzer at the resolution of 120 K. MS/MS tandem mass spectra were generated in the ion trap mass analyzer by collision-induced dissociation of peptide ions at a normalized collision energy of 35%. The resulting MS/MS spectra were searched against human gamma D protein database using Mass Matrix software (MassMatrix, Inc) with the mass errors of 10 ppm and 0.8 Da for MS1 and MS/MS scans, respectively. The oxidation of methionine was set for variable modifications, and three miscleavages were allowed for the search.

### Molecular docking

To investigate the binding location, binding affinity (kilocalorie/mole), and molecular interactions of lead compounds to HγD, the X-ray crystal structure at 1.25 A resolution (Protein Data Bank [PDB] ID: 1HK0) (43) was retrieved from PDB. We utilized molecular docking *via* AutoDock Vina3 (83) within UCSF Chimera 1.14.4. Lead compound structures were obtained *via* PubChem. Conversion from .SDF file format to .PDB file format and ligand preparation was completed within BIOVIA Discovery Studio Visualizer 2020 by Dassault Systèmes.

Within AutoDock Vina3, docking parameters included exhaustiveness settings at 8. Blind docking (defined as HγD contained within the three-dimensional grid box for molecular docking) of the entire surface of HγD was performed to determine general binding locations and affinities.

Postdocking analysis was performed within Discovery Studio Visualizer 2020 to view specific binding interactions of candidate drugs to HγD, such as hydrogen bonds, van der Waals interactions, and other electrostatic interactions. Interacting residues from HγD with drugs were recorded in a Microsoft Excel spreadsheet for proper viewing of patterns and binding affinities among lead compounds that warranted further investigation.

To investigate specific binding residues to HγD and the human MAC (^70^KFVIFLDVKHFSPEDLTVK^88^) as detailed by Banerjee *et al.* (53), we utilized the external protein–protein web-docking server ZDOCK 3.0.26 because of ligand limitations (maximum rotatable bonds up to 32) within AutoDock Vina (84).

The aforementioned methodology for molecular docking was performed for both human and mouse gamma D crystallins (MγD) (SMR ID: P04342) (84) retrieved from the SWISS-MODEL repository (85) as well as for human (HγS) (PDB ID: 6FD8) (86) and mouse gamma S crystallins (MγS) (PDB in Europe [PDBe] ID: 1ZWM) (87) retrieved from PDB2 of their X-ray crystal structure at 2.10 Å and NMR structure, respectively.

### Impact of protein concentration on the reproducibility of the drug data

Comparative studies were carried out in which total bovine γ-crystallins (BGC) (pool 4), HγD, and MγS were incubated at varying protein concentrations (from 0.06 mg/ml to 2.0 mg/ml buffer A, *i.e.* 50 mM K^+^/PO_4_, pH 7.2) with 400 μM drug C or G in DMSO, as well as drug A (agaric acid) in lieu of drug B (bixin) whose stock was exhausted. Final DMSO concentration was 5% including in control samples. Microtiter plates were first incubated for 10 min at 72 °C and reincubated for 10 more min. Absorbance was measured at both 10 and 20 min total incubation time. The results (Fig. S7) show that aggregation propensity and prevention by the drugs was strongly dependent on protein concentration. Both drugs C and G reproducibly suppressed absorbance of BGC at 2.0 mg/ml at 10 and 20 min but surprisingly stimulated aggregation at lower concentrations at both times. At 10 min but not at 20 min, drug A improved BGC aggregation at 2.0 mg/ml. Similarly at low protein, drugs C and G but not A worsened HγD aggregation. At 2.0 mg/ml and 20 min, baseline increase of control protein was suppressed by drug A, whereas no baseline change was noted for drugs C and G. MγS was highly destabilized by heat treatment as a function of protein concentration, and all drugs (C, G, and A) blocked absorbance above 0.5 mg/ml. These data emphasize the need to carefully optimize experimental conditions as a function of γ-crystallin species and type of drug under consideration.

### Rigor and reproducibility

While this study was under review, we became aware of the fact that testing of drug C supplied to an outside laboratory showed worsening rather than improved prevention of heat-mediated aggregation when HγD was incubated with 600 μM drug C. To clarify this discrepancy, we performed a comparative study on the ability of 400 μM drug C, G, and A to inhibit solution turbidity at 600 nm as a function of protein concentration. Overall results indicate that turbidity development and ability of the drugs to suppress it is reproducible but only at high protein concentration. We also found that initial turbidity occasionally develops in the presence of drugs but tends to disappear upon heating, suggesting either improved solubilization of the drug by the protein or reversal of its aggregated state. For specific experimental details, see Experimental procedures section.

## Data availability

All the data described are located within the article and the online supporting information, except for the ^15^N–^1^H NMR spectra on drug C with and without HγD interaction (negative) and the computer generated list of all tryptic peptides resulting from the trypsin digestion of HγD incubated with drug G, both of which can be obtained upon request from the corresponding author.

## Supporting information

This article contains supporting information (88).

## Conflict of interest

V. M. M. is on the Scientific Advisory Board of Revel Pharmaceuticals. Inc. All other authors declare that they have no conflicts of interest with the contents of this article.

## Footnotes

† Deceased.
