# Supplementary material for: α-Crystallin chaperone mimetic drugs inhibit lens γ-crystallin aggregation: Potential role for cataract prevention
Source: J Biol Chem. 2022 Aug 28;298(10):102417. doi: 10.1016/j.jbc.2022.102417 (PMC9525908; doi:10.1016/j.jbc.2022.102417)
Supplement: Supporting information [file mmc1.pdf]

Supporting Information for

α-Crystallin chaperone mimetic drugs inhibit lens γ-crystallin aggregation: potential role for cataract prevention

Sidra Islam[1], Michael Do[1], Brett S. Frank[1], Grant L. Hom[1], Samuel Wheeler[2], Hisashi Fujioka[3], Benlian Wang[4] , Geeta Minocha[1], David R Sell[1], Xingjun Fan[5], Kirsten J. Lampi[2], Vincent M. Monnier[1, 6]
1Dept of Pathology, Case Western Reserve University, Cleveland, OH 44106,
2Dept of Integrative Biosciences, Oregon Health & Sciences University, Portland, OR 97239,
3Cryo-EM Core Facility, School of Medicine, Case Western Reserve University, Case Western Reserve University, Cleveland, OH 44106,
4Center for Proteomics, Dept. of Nutrition, Case Western Reserve University, Cleveland, OH 44106
5Dept of Cell Biology and Anatomy, Augusta University, Georgia, GA 30912,
6Dept of Biochemistry, Case Western Reserve University, Cleveland OH 44106

Content:
Table S1. Ranking of the top six drug candidates by efficacy of turbidity suppression based on mean of all experiments in Table II.
Table S2. Radius of the aggregates of WT-HγD with and without drug C, G and M at the end point of thermal stress (72 °C, 20 min) as observed from DLS spectra
Table S3. Percent inhibition in the binding of bis-ANS by drugs in HγD, MγS and OPJ- MγS
Table S4. List of primers used for making WT-HγD mutants
Table S5. Percent inhibition in heat mediated aggregation of WT-MγS and OPJ-MγS by 500 µM of candidate inhibitor drugs at the end point of turbidity assay (72 °C, 20 min)
Table S6. Radius of the aggregates of WT-MγS with and without drug C, G and M at the end point of thermal stress (72 °C, 20 min) as observed from DLS spectra
Table S7. Radius of the aggregates of OPJ-MγS with and without drug C, G and M at the end point of thermal stress (72 °C, 20 min) as observed from DLS spectra
Table S8. Melting temperatures of WT-MγS and OPJ- MγS with and without drug C, G and M calculated from the thermal unfolding curves. N-ter and C-ter refer to N- and C-terminal domain
Table S9. Trypsin miscleavage sites detected in HγD incubated with Drug G compared to no drug treatment
Table S10. Summary of screen compounds whose activity either worsened or improved the H_2O_2 mediated aggregation of bovine total gamma crystallins with or without added thermal stress
Figure S1. Representative Sephadex G-200 gel filtration profile of bovine lens extract and effect of concentration on the ability of candidate drugs to inhibit thermal induced aggregation of recombinant human γD crystallin (WT-HγD) and bovine γ-crystallin-rich Pool 4
Figure S2. SDS-Page and western blot of purified WT-HγD and its mutants W43R, R58H, R14C, and far- and near-UV CD spectra of WT-HγD with and without drugs
Figure S3. SDS-Page and western blot of WT-MγS and its mutant OPJ-MγS
Figure S4. Effects of Closantel, Gambogic acid and other drugs on the biophysical and morphological properties of total mouse gamma crystallins, WT-MγS and its mutant OPJ-MγS exposed to oxidative and thermal stress
Figure S5. Fluorescence emission spectra, Stern-Volmer plot and modified Stern-Volmer plot of tryptophan fluorescence quenching of WT-HγD
Figure S6. Fluorescence emission spectra, b) Stern-Volmer plot and c) modified Stern-Volmer plot of tryptophan fluorescence quenching of WT-MγS
Figure S7. Role of the protein concentration on crystallin aggregation and its potential suppression by Drugs C, G and A: a warning

Table S1: Ranking of the top six drug candidates by efficacy of aggregation suppression based on mean of all experiments in Table II.

Drug	% suppression (n=9)	(%) mean +/- SD
C	60/73/65/73/71/77/70/52/76	68.6 8.2
G	48/75/69/75/46/73/55/48/89	64.2 15.4
X	30/55/61/47/68/60/60/50/71	55.8 12.3
D	40/44/66/78/61/76/59/30/32	54.0 18.1
B	23/54/63/60/60/51/57/25/46	48.8 14.9
M	40/16/38/73/39/48/55/25/46	42.2 16.5

Note: Drug T appeared only once among the top six candidates and was not further considered.

Table S2: Radius of the aggregates of WT-HγD with and without drug C, G and M at the end point of thermal stress (72 °C, 20 min) as observed from DLS spectra

Sample	Radius (nm)			Percent Mass		
	Peak 1	Peak 2	Peak 3	Peak 1	Peak 2	Peak 3
CON (No Heat)	2.3	9.4	51	99.2	0.7	0.1
CON (Heat)	22.9	409.1	7261	2.6	19.2	78.3
C	30.5	-	-	100	-	-
G	84.9	-	-	100	-	-
M	10.9	568.5	-	12.7	87.3	-

Table S3: Percent inhibition of binding of bis-ANS to HγD, MγS and OPJ- MγS by Drugs

Drugs	WT-HγD	WT-MγS	OPJ-MγS
M	4.28±0.0003	1.95±0.0017	3.48±0.0010
X	15.16±0.0010	10.17±0.0014	2.45±0.00075
B	27.86±0.0009	11.94±0.0018	11.83±0.00091
D	34.5±0.0006	19.49±0.0018	7.76±0.00097
C	43.82±0.0012	27.16±0.0011	20.28±0.00089
G	44.64±0.0011	40.72±0.0008	31.62±0.00057

Table S4: List of primers used for making WT-HγD mutants

**Mutants**	**Forward Primer**	**Reverse Primer**
W43R	5'- CGGCTGCCGCATGCTCTATGAGCAGCCC -3'	5'-AGCATGCGGCAGCCGCTGTCCACGCG-3'
R14C	5'-CCAGGGCTGCCACTATGAATGCAGCAGCGACC-3'	5'-TAGTGGCAGCCCTGGAAGCCCCGGTC-3'
R58H	5'-CTTCCTGCACCGCGGCGACTATGCCGACC-3'	5'-CCGCGGTGCAGGAAGTACTGGAGGCCCG-3'

Table S5: Percent inhibition of heat mediated aggregation of WT-MγS and OPJ-MγS by 500 μM of selected drugs at the end point of turbidity assay (72 °C, 20 min)

**Drugs**	**Percent Inhibition**	
	**WT-MGS**	**OPJ-MGS**
C	80.5±1.87	82.0±0.49
G	80.5±2.45	91.6±0.09
X	39.7±3.42	60.9±0.80
D	80.4±0.44	85.1±0.68
B	55.5±8.03	77.6±0.21
M	46.3±4.23	33.6±2.36

Table S6: Radius of the aggregates of WT-MγS with and without drug C, G and M at the end point of thermal stress (72 °C, 20 min) as observed from DLS spectra

**Sample**	**Radius (nm)**		**Percent Mass**	
	Peak 1	Peak 2	Peak 1	Peak 2
CON (No Heat)	2.1	9.0	92.2	7.8
CON (Heat)	5789.7	-	100	-
C	86.6		100	-
G	77.9	-	100	-
M	622.0	6200.7	1.4	98.6

Table S7: Radius of the aggregates of OPJ-MγS with and without drug C, G and M at the end point of thermal stress (72 °C, 20 min) as observed from DLS spectra

**Sample**	**Radius (nm)**		**Percent Mass**	
	**Peak 1**	**Peak 2**	**Peak 1**	**Peak 2**
CON (No Heat)	1.9	7.4	95.2	4.8
CON (Heat)	2400.8	-	100	-
C	21.9	358.7	32	68
G	79.3	-	100	-
M	53.1	5594.1	0	100

Table S8: Melting temperatures of WT-MγS and OPJ- MγS with and without drug C, G and M calculated from the thermal unfolding curves. N-ter and C-ter refer to N- and C-terminal domain

**Drug**	**Tm (°C)**		
	**WT-MGS**	**OPJ-MGS**	
		N-ter	C-ter
Con	70	48	63
C	72	50	69
G	73	50	70
M	68	48	63

Table S9: Trypsin miscleavage sites detected in HγD (2.5mg/ml) incubated with 400uM Drug G with 5%DMSO for 20 min at 72°C compared to no drug treatment with 5% DMSO.

Number	Peptide	Site	Mw(obs)	Mw(cal)	Error (Da)
4-15	ITLYEDRGFQGR	R10	1454.7369	1454.7387	-0.0018
60-77	RGDYADHQQWMGLSDSVR	R60	2120.9566	2120.9567	-0.0001
78-89	SCRLIPHSGSHR	R80	1349.6838	1349.6855	-0.0018
118-142	FNEIHSLNVLEGSWVLYELSNYRGR	R140	2955.5072	2995.5061	0.0011
118-153	FNEIHSLNVLEGSWVLYELSNYRGR-QYLLMPGDYR	R140, R142	4232.1028	4232.1022	0.0006
154-174	YQDWGATNARVGSLRRVIDFS	R168, R169	2411.2218	2411.2215	0.0004

Table S10: Summary of screened compounds whose activity either worsened or improved the turbidity of bovine gamma crystallins exposed to H_2O_2 with or without added thermal stress

Drugs found to worsen solution turbidity at 600 nm (N=241)
Drugs found to mildly improve solution turbidity at 600 nm (n=105)
Drugs found to more strongly improve solution turbidity at 600nm when 10 min heat shock at 72ºC is added (N=30)

MOLENAME	Tradename	cas#	Bioactivity
0ISOPIMPINELLIN		482-27-9	antineoplastic, antifungal, antviral, insect antifeedant
1,4-NAPHTHOQUINONE		130-15-4	antibacterial, antineoplastic
1-HYDROXY-3,6,7-TRIMETHOXY-2,8-DIPRENYLXANTHONE		15404-76-9	
2',4'-DIHYDROXY-4-METHOXYCHALCONE		81674-91-1	
2,4-DIMETHOXY-2'-HYDROXYCHALCONE		32272-18-7	PGE2 inhibitor

2-MERCAPTOBENZOTHIAZOLE		149-30-4	antibacterial, antifungal. inhibits dopamine beta-hydroxylase
2-METHOXYRESORCINOL		29267-67-2	
3,4',5,6,7-PENTAMETHOXYFLAVONE		4472-73-5	
3,4'-DIHYDROXYFLAVONE		14919-49-4	
3,4'-DIMETHOXYFLAVONE			
3',6-DIHYDROXYFLAVONE		71592-46-6	
3,7-DIHYDROXYFLAVONE		492-00-2	
3,7-DIMETHOXYFLAVONE		20950-52-1	
3-beta-ACETOXYALLOPREG-16-EN-20-ONE			
3beta-HYDROXYCHOL-5-ENIC ACID		5255-17-4	
3-HYDROXYFLAVONE		577-85-5	
4-ACETOXYPHENOL		3233-32-7	antioxidant
4'-METHOXYFLAVONE		4143-74-2	
4-NONYLPHENOL		104-40-5	weevil pheromone, shows estrogenic activity
5,4'-DIMETHOXY-7-HYDROXYFLAVONE			
6,2'-DIMETHOXYFLAVONE			
6,3'-DIMETHOXYFLAVONE		79786-40-6	
6-HYDROXYFLAVONE		6665-83-4	anxiolytic
7,4'-DIMETHOXYISOFLAVONE			
7-DEACETYLKHIVORIN			
7-DESACETOXY-6,7-DEHYDROGEDUNIN			
7-DESHYDROXYPYROGALLIN-4-CARBOXYLIC ACID			GPR-35 agonist
ABIETIC ACID		514-10-3	antiinflammatory, testosterone 5a reductase inhibitor
ACEDAPSONE	HANSOLAR	77-46-3	antimalarial, leprostatic
ACETOXOLONE			antiulcer
ACETYL ISOGAMBOGIC ACID			
ACITRETIN	SORIATANE	55079-83-9	antipsoriatic
ACRIFLAVINIUM HYDROCHLORIDE	PANFLAVIN	8063-24-9	antiinfective, intercalating agent
ACRISORCIN	AKRINOL	7527-91-5	antifungal
ADRENALONE HYDROCHLORIDE	STRYPHNASAL	62-13-5	adrenergic (opthalmic)
AGARIC ACID		666-99-9	antiperspirant
AGELASINE			cytotoxic, antineoplastic; mixed isomers
ALBENDAZOLE	ALBENZA	54965-21-8	anthelmintic
ALEURETIC ACID		533-87-9	
ALGINIC ACID [Mol Wt ~200,000; monomers shown]		9005-32-7	emulsifying agent
ALLYLESTRENOL		432-60-0	progestogen
ALLYLTHIOUREA		109-57-9	chelating agent
alpha-MANGOSTIN		6147-11-1	antifungal, antilarval
alpha-TOCHOPHERYL ACETATE [4mM]	EPHYNAL	58-95-7	vitamin E
alpha-TOXICAROL (dl)		82-09-7	pysciotoxin
AMINACRINE	MONACRIN	90-45-9, 134-50-9 [aminacrine hydrochloride]	local antiseptic
AMINOETHYLISOTHIOUREA DIHYDROBROMIDE		56-10-0	NOS inhibitor
AMIODARONE HYDROCHLORIDE	CORDARONE	1951-25-3	adrenergic agonist, coronary vasodilator, Ca channel blocker
AMITRIPTYLINE HYDROCHLORIDE	ELAVIL	549-18-8	antidepressant

AMODIAQUINE DIHYDROCHLORIDE	CAMOQUIN	6398-98-7, 69-44-3 [anhydrous], 86-42-0 [amodiaquine]	antimalarial
AMOXAPINE	ASENDIN	14028-44-5	antidepressant, inhibits norepinephrine uptake
ANTHOTHECOL		10410-83-0	antimalarial
ANTIAROL		642-71-7	
ANTIMONY POTASSIUM TARTRATE TRIHYDRATE		28300-74-5	antischistosomal
APIOLE		523-80-8	antipyretic, diuretic, insecticide
APIXABAN	ELIQUIS	503612-47-3	Factor Xa inhibitor, anticoagulant, antithrombotic
APOMORPHINE HYDROCHLORIDE	APOKYN	41372-20-7, 314-19-2 [anhydrous], 58-00-4 [apomorphine]	dopamine receptor agonist, antiparkinsonian, erextile dysfunction therapy
APRAMYCIN SULFATE		65710-07-8	antibacterial; LD50(iv) 280mg/kg(mouse)
ARACHIDONIC ACID		506-32-1	stimulates NO biosynthesis, vasodilator
ARTHONOIC ACID		25556-24-5	
ASCORBYL PALMITATE	EMPROVE	137-66-6	antioxidant
ATAZANAVIR SULFATE	REYATAZ	229975-97-7	antiviral, HIV protease inhibitor
ATORVASTATIN CALCIUM	LIPITOR	134523-03-8	antihyperlipidemic, HMGCoA reductase inhibitor
ATOVAQUONE	MEPRON	95233-18-4	antipneumocystic, antimalarial
AURIN TRICARBOXYLIC ACID		4431-00-9	endonuclease inhibitor; apoptosis inhibitor, topoisomerase II inhibitor
AVOBENZONE	PARSOL	70356-09-1	sunscreen
AVOCADENE		24607-08-7	antifungal
AVOCADENE ACETATE		24607-09-8	antifungal, plant growth inhibitor
AVOCADENOFURAN		25346-24-1	
AVOCADYNE ACETATE		24607-06-5	antifungal
AZTREONAM	AZACTAM	78110-38-0	antibacterial
BAZEDOXIFENE ACETATE		198481-33-3	antiosteoporotic
BENDAMUSTINE HYDROCHLORIDE	RIBOMUSTINE	3543-75-7	antineoplastic
BENSERAZIDE HYDROCHLORIDE	MADOPA	322-35-0	decarboxylase inhibitor
BENZALKONIUM CHLORIDE HYDRATE	ROCCAL	8001-54-5	antiinfective (topical)
BENZBROMARONE	DESURIC	3562-84-3	uricosuric
BENZETHONIUM CHLORIDE	PHEMERIDE	121-54-0	antiinfective (topical)
BENZYDAMINE HYDROCHLORIDE	TANTUM	132-69-4	analgesic, antipyretic, antiinflammatory
BERBAMINE HYDROCHLORIDE		6078-17-7	antihypertensive, skeletal muscle relaxant
beta-CAROTENE [2mM]	SOLATENE	7235-40-7	antioxidant; provitamin A
BETULIN		473-98-3	antineoplastic, antihyperlipidemia
BILIRUBIN		635-65-4	antioxidant
2',2'-BISEPIGALLOCATECHIN DIGALLATE			
BITHIONOL	LOROTHIDOL	97-18-7	anthelmintic, antiseptic
BIXIN		39937-23-0	antiineoplastic
BRAZILEIN		600-76-0	antineoplastic, induces apoptosis, antiinflammatory
BRAZILIN		474-07-7	inhibits platelet aggregation, PLA2, PKC, protein phosphatase, insulin receptor kinase, nitric oxide synthase, antineoplastic
BRONOPOL	BRONOSOL	52-51-7	antiinfective
BROXALDINE		3684-48-6	antiinfective, antifungal

BRUCINE		4845-99-2, 357-57-3 [brucine]	central stimulant
BUSSEIN		41060-14-4	
CABAZITAXEL	JEVTANA	183133-96-2	antineoplastic
CANDESARTAN CILEXTIL	ATACAND	139481-59-7	angiotensin 1 receptor antagonist
CANDICIDIN	VANOBID	1403-17-4	antifungal
CANRENONE		976-71-6	aldosterone antagonist; antifibrogenic
CAPTAN		133-06-2	fungicide, antibacterial
CARAPIN		3463-88-5	
CARAPIN-8(9)-ENE			
CARBAZOCHROME	ADRENOXYL	69-81-8	hemostatic agent
CARBENOXOLONE SODIUM	SANODIN	7421-40-1, 5697-56-3 [carbenoxolone]	antiinflammatory, antisecretory, antiulcer
CARBIMAZOLE		22232-54-8	antithyroid
CARVEDILOL	COREG	72956-09-3	beta-adrenergic blocker
CARVEDILOL PHOSPHATE	COREG CR	610309-89-2	antianginal, antihypertensive
CEDRELONE		1254-85-9	
CEFTAZIDIME	TAZIDIME	72558-82-8	antibacterial
CEFUROXIME SODIUM	ZINACEF	56238-63-2	antibacterial
CELASTROL		34157-83-0	antineoplastic, NO synthesis inhibitor, chaperone stimulant
CELECOXIB	CELEBREX	169590-42-5	antiarthritic, cyclooxygenase2 inhibitor
CEPHALOTHIN SODIUM	KEFLIN	58-71-9, 153-61-7 [cephalothin]	antibacterial
CETALKONIUM CHLORIDE	ZETTYN	122-18-9	antinfective
CETYLPYRIDINIUM CHLORIDE	CEPACOL	6004-24-6, 123-03-5 [anhydrous]	antiinfective (topical)
CHAULMOOGRIC ACID		502-30-7	antibacterial (mycobacteria), antileprotic
CHENODIOL	CHENIX	474-25-9	anticholithogenic, antilipemic agent
CHICAGO SKY BLUE		2610-05-1	inhibitor of aminoacid uptake
CHINIOFON	YATREN	8002-90-2	antiprotozoal, amebicide
CHLORAMPHENICOL PALMITATE	CHLOROPAL	530-43-8	antibacterial, antirickettsial
CHLORAZANIL HYDROCHLORIDE	DAQUIN	2019-25-2	diuretic
CHLORHEXIDINE DIHYDROCHLORIDE	LISIUM	3697-42-5, 55-56-1 [chlorhexidine]	antibacterial (topical), disinfectant
CHLORMIDAZOLE		3689-76-7	antifungal
CHLOROPHYLLIDE Cu COMPLEX Na SALT	CHLORESIUM	11006-34-1	antineoplastic
CHLOROTHALONIL		1897-45-6	antifungal, pesticide, acaricide
CHLORPROMAZINE HYDROCHLORIDE	THORAZINE	69-09-0	antiemetic, antipsychotic
CHOLECALCIFEROL		67-97-0	vitamin D3
CHRYSENEQUINONE		100900-16-1	cytotoxic, antineoplastic, aldehyde dehydrogenase inhibitor
CHRYSIN		480-40-0	diuretic
CHRYSIN DIMETHYL ETHER		21392-57-4	
CISPLATIN	PLATINOL	15663-27-1	antineoplastic, convulsant
CLINDAMYCIN PALMITATE HYDROCHLORIDE	CLEOCIN	25507--04-4	antibacterial, inhibits protein synthesis
CLIOQUINOL	VIOFORM	130-26-7	antiseptic, antiamebic
CLOFAZIMINE	LAMPRENE	2030-63-9	antibacterial, antilepretic, antituberculosis
CLOFOCTOL	OCTOPHENE	37693-01-9	antibacterial
CLOPERASTINE HYDROCHLORIDE	NOTUSSIL	3703-76-2	antitussive
CLOSANTEL	FLUKIVER	57808-65-8	anthelmintic
CURCUMIN		458-37-7	antiedemic, antiinflammatory, bile stimulant; antibacterial,

CYCLOSPORINE	SANDIMMUNE	59865-13-3	antifungal, lipo/cyclooxygenase inhibitor immunosuppressant
CYPROHEPTADINE HYDROCHLORIDE	PERIACTIN	969-33-5	H1-antihistamine, antipruritic
CYSTAMINE DIHYDROCHLORIDE		56-17-7	hepatoprotectant, t-glutaminase inhibitor, heat shock protein promoter, caspase inhibitor
CYSTEAMINE HYDROCHLORIDE		60-23-1	antiurolithic, depigmentation, radiation protectant
DANTHRON	DORBANE	117-10-2	cathartic
DANTROLENE SODIUM	DANTRIUM	24868-20-0, 14663-23-1 [anhydrous], 7261-97-4 [dantrolene]	muscle relaxant (skeletal)
DEACETYLGEDUNIN			
DECOQUINATE [5mM]		18507-89-6	coccidiostat
DEGUELIN(-)		522-17-8	antineoplastic, antiviral, insecticide, ornithine decarboxylate inhibitor
DEMETHYLNOBILETIN		2174-59-6	
DEOXYGEDUNIN		21963-95-1	neuroprotective, anti-depressant. learning enhancement
DEQUALINIUM CHLORIDE	FLUOMIZIN	522-51-0, 6707-58-0 [dequalinium]	antiinfectant, antineoplastic
DESLORATIDINE	CLARINEX	100643-71-8	H1-antihistamine
DIACERIN		13739-02-1	antiinflammatory
DICHLORISONE ACETATE	DILODERM	79-61-8	antipruretic
DICLAZURIL	CLINACOX	101831-37-2	coccidiostat
DICLORALUREA		116-52-9	antibacterial
DICTAMNINE		484-29-7	antifungal, vasal relaxant, smooth muscle contractant
DIFFRACTAIC ACID		436-32-8	antiproliferative
DIFLUBENZURON	ECTOGUARD	35367-38-7	insecticide, inhibits melanosome synthesis
DIHYDROATRANORIN		69168-12-3	
DIHYDROCELASTROL			
DIHYDROCELASTRYL DIACETATE			chaperone stimulant
DIHYDROFISSINOLIDE			
DIHYDROGAMBOGIC ACID			
DIHYDROGEDUNIN			
DIHYDROROTENONE		6659-45-6	acaricide, ectoparasiticide, antineoplastic, mitochondrial poison, binds tubulin
DIMESNA		16208-51-8	antineoplastic, uroprotectant
DIPERODON HYDROCHLORIDE		537-12-2	analgesic, anesthetic
DOBUTAMINE HYDROCHLORIDE	DOBUTREX	49745-95-1, 34368-04-2 [dobutamine]	cardiotonic
DOCUSATE SODIUM	DOC-Q-LACE	577-11-7	stool softener
DOPAMINE HYDROCHLORIDE	INTROPIN	62-31-7, 51-61-6 [dopamine]	cardiotonic, antihypotensive
DROPROPIZINE		17692-31-8	antitussive
DROXYDOPA	DOPS	23651-95-8	neurogenic hypotension, antiparkinsonian
ECONAZOLE NITRATE	SPECTAZOLE	68797-31-9, 27220-47-9 [econazole]	antifungal
EMODIN		518-82-1	antibacterial, antineoplastic, cathartic, tyrosine kinase inhibitor
ENOXACIN	PENETREX	74011-58-8	antibacterial
ENOXOLONE		471-53-4	antitussive, antiinflammatory, antibacterial
EPIANDROSTERONE		481-29-8	weak androgen

EPIGALLOCATECHIN-3-MONOGALLATE		989-51-5	
ERDOSTEINE	SECRESOLV	84611-23-4	mucolytic
ERGOCALCIFEROL	DRISDOL	50-14-6	antirachitic vitamin, Vitamin D2
ERYTHROMYCIN ESTOLATE	ILOSONE	134-36-1, 114-07-8 [erythromycin]	antibacterial
ERYTHROSINE SODIUM		16423-68-0	color additive
ESCIN		6805-41-0	membrane permeabilizer
ESTROPIPATE	OGEN	7280-37-7	estrogen
ETHACRIDINE LACTATE	RIVANOL	1837-57-6; 6402-23-9 (hydrate)	antiseptic, abortifaceant
ETHACRYNIC ACID	EDECRIN	58-54-8	diuretic
ETHANOLAMINE OLEATE	ETHAMOLIN	2272-11-9	sclerosing agent
ETHAVERINE HYDROCHLORIDE	ETHAQUIN	985-13-7, 486-47-5 [base]	antispasmodic
ETHISTERONE		434-03-7	progestogen
ETHYNODIOL DIACETATE		297-76-7	progestin
EVANS BLUE		314-13-6	glutamate uptake inhibitor, AMPA blocker
FAST GREEN FCF		2353-45-9	carcinogen, mutagen, inhibitor of synaptic activity, immune suppressant
FLAVONE		525-82-6	
FLUVASTATIN SODIUM	LESCOL	93957-55-2	antihyperlipidemic, HMGCoA reductase inhibitor
FORMONONETIN		485-72-3	phytoestrogen
FUMARPROTOCETRARIC ACID		489-50-9	antibacterial
GALLAMINE TRIETHIODIDE	FLAXADIL	65-29-2, 153-76-4 [gallamine]	muscle relaxant (skeletal)
GALLIC ACID		149-91-7	antineoplastic, astringent, antibacterial
GAMBOGIC ACID		2752-65-0	antiinflammatory, cytotoxic, inhibits HeLa cells in vitro;
GARCINOLIC ACID		91893-83-3	antineoplastic, neuraminidase inhibitor
GARDENIN B		2798-20-1	
GEDUNOL			
GLIQUIDONE	GLURENORM	33342-05-1	antidiabetic
GOSSYPOL		303-45-7	antispermatogenic, antineoplastic, antiHIV
GOSSYPOL-ACETIC ACID COMPLEX			male contraceptive, dehydrogenase inhibitor, antimalarial
GRAMICIDIN (gramicidin A shown)		1405-97-6	antibacterial
GUANFACINE HYDROCHLORIDE	TENEX	29110-48-3	antihypertensive
HAEMATOPORPHYRIN		14459-29-1	antidepressant, antineoplastic
HAEMATOXYLIN		517-28-2	
HAEMATOXYLIN PENTAACETATE			
HARMANE		486-84-0	intercalating agent, sedative
HARMOL			antineoplastic, apoptosis inducer
HEMATEIN		475-25-2	CK2 inhibitor, antineoplastic, TNFa inhibitor, antiinflammatory
HETEROPEUCENIN, METHYL ETHER		26213-95-6	
HEXACHLOROPHENE	PHISOHEX	70-30-4	antiinfective (topical)
HEXETIDINE	STERISIL	141-94-6	antifungal
HOMIDIUM BROMIDE		1239-45-8	antiprotozoal, intercalcates with DNA
HOMOSALATE	EUSOLEX	118-56-9	UV screen, analgesic
HYCANTHONE		3105-97-3	anthelmintic, hepatotoxic
HYDROQUINONE	SOLAQUIN FORTE	123-31-9	depigmentor, antioxidant
ICARIIN		489-32-7	hepatoprotective

IDEBENONE		58186-27-9	cognition enhancer, nootropic
IDOXURIDINE	DENDRID	54-42-2	antiviral
INDOLE-3-CARBINOL		700-06-1	antineoplastic; inhibitor of Amyloid-beta deposition
INDOXYL ACETATE			plant growth promoter
IODOFORM	Kri1	75-47-8	antiseptic, disinfectant
IRIGENOL HEXAACETATE			
ISOCONAZOLE NITRATE	FAZOL	24168-96-5	antibacterial, antifungal
ISOFLUPREDONE ACETATE	PREDEF	338-98-7	antiinflammatory
ISOOSAJIN		5745-54-0	
ISOPOMIFERIN			antioxidant
ISOPROPAMIDE IODIDE	DARBID	71-81-8, 7492-32-2 [isopropamide]	anticholinergic
ITRACONAZOLE HYDROCHLORIDE	SPORANOX	84625-61-6	antifungal
IZALPININ		480-14-4	inhibits bladder contractility
JUGLONE		481-39-0	antineoplastic, antifungal, antioxidant, Pin 1 inhibitor
KOPARIN		65048-75-1	antiinflammatory
LACCAIC ACID A		15979-35-8	inhibits DNA methyltransferase I
LEVODOPA	LARODOPA	59-92-7	antiparkinsonian
LEVOFLOXACIN	LEVAQUIN	138199-71-0	antibacterial
LITHOCHOLIC ACID		434-13-9	LD50(mouse) 3900 mg/kg po
LOBARIC ACID		522-53-2	antibacterial
LOMERIZINE HYDROCHLORIDE	TERRANAS	101477-55-8	antimigraine, cerebral vasodilator, Ca channel blocker
LOPERAMIDE HYDROCHLORIDE	IMODIUM	34552-83-5, 53179-11-6 [loperamide]	Ca channel blocker
LOPINAVIR		192725-17-0	antiviral; HIV protease inhibitor
LORGLUMIDE SODIUM		97964-56-2 (acid)	CCK receptor antagonist
MANGOSTIN TRIMETHYL ETHER			
MECLOFENAMATE SODIUM	MECLOMEN	6385-02-0	antiinflammatory, antipyretic
MEFLOQUINE HYDROCHLORIDE	LARIAM	53230-10-7	antimalarial
MENAQUINONE-4			antioxidant, alkaline phosphatase enhancer
MENTHYL BENZOATE		71617-14-6	
MESALAMINE	ASACOL	89-57-6	antiinflammatory
MESTRANOL		72-33-3	estrogen, with progesterone as oral contraceptive
METERGOLINE		17692-51-2	analgesic, antipyretic
METHACYCLINE HYDROCHLORIDE	RONDOMYCIN	3963-95-9, 914-00-1 [methacycline]	antibacterial
METHIMAZOLE	TAPAZOLE	60-56-0	antihyperthyroid
METHYL 7-DESHYDROXYPYROGALLIN-4-CARBOXYLATE		77-41-8	
METHYL GAMBOGATE METHYL ETHER			
METHYLBENZETHONIUM CHLORIDE	DELAVAN	1320-44-1, 25155-18-4 [anhydrous]	antiinfective
METHYLENE BLUE		7220-79-3	antimethemoglobinemic, cyanide antidote
METITEPINE MESYLATE		20229-30-5	5HT1&2 receptor antagonist
METRONIDAZOLE	FLAGYL	443-48-1, 69198-10-3 [metronidazole hydrochloride]	antiprotozoal
MINOCYCLINE HYDROCHLORIDE	SOLODYN	13614-98-7, 10118-90-8 [minocycline]	antibacterial
MITOXANTRONE HYDROCHLORIDE	NOVANTRONE	70476-82-3, 65271-80-9 [base]	antineoplastic
MONTELUKAST SODIUM	SINGULAIR	151767-02-1	leucotriene antagonist, antiasthmatic
MUNDULONE		481-94-7	antineoplastic

MYRICETIN		529-44-2	antiHIV, topoisomerase II inhibitor
NAFTOPIDIL	AVISHOT	57149-07-2	antihypertensive, alpha-blocker, 5HT1a agonist
NICLOSAMIDE	NICLOSIDE	50-65-7	anthelmintic, teniacide
NILVADIPINE	ESCOR	75530-68-6	antihypertensive, antianginal
NITARSONE	HISTOSTAT	98-72-6	antiprotozoal
NITAZOXANIDE	ALINIA	55981-09-4	antiparasitic
NITROXOLINE	ENTEROCOL	4008-48-4	antibacterial
NORGESTIMATE		35189-28-7	progestin
NORHARMAN		244-63-3	plant growth inhibitor; mutagen
NORTRIPTYLINE HYDROCHLORIDE	AVENTYL	894-71-3, 72-69-5 [nortriptyline]	antidepressant
NYSTATIN	MYCOSTATIN	114-90-9	antifungal, binds to membrane sterols
OBTUSAQUINONE		21105-15-7	antifungal, antineoplastic
OCTINOXATE		5466-77-3	sunscreen, UV-B absorber
OCTOCRYLENE	EUSOLEX	6197-30-4	sunscreen
OLTIPRAZ		64224-21-1	schistosomicide, antineoplastic
OMEGA-3-ACID ESTERS (EPA shown)	LOVAZA	86227-47-6	hypolipidemic
OSAJIN		482-53-1	antioxidant, cardioprotectant
OXICONAZOLE NITRATE	OXISTAT	64211-46-7	antifungal
OXIDOPAMINE HYDROCHLORIDE		28094-15-7	adrenergic agonist (opthalmic)
OXYCLOZANIDE	ZANIL	2277-92-1	anthelmintic
OXYPHENONIUM BROMIDE	ANTRENYL	50-10-2	anticholinergic, anticonvulsant
OXYTETRACYCLINE	TERRAMYCIN	6153-64-6, 79-57-2 [anhydrous]	antibacterial
PAROXETINE HYDROCHLORIDE	PAXIL	78246-49-8, 61869-08-7(base)	antidepressant
PATULIN		149-29-1	antibacterial, antitumor
PAZUFLOXACIN MESYLATE	PAZUCROSS	163680-77-1; 127045-41-4	antibacterial
PENFLURIDOL	SEMAP	26864-56-2	antipsychotic
PERHEXILINE MALEATE	PEXID	6724-53-4, 6621-47-2 [perhexiline]	coronary vasodilator
PERSEITOL		527-06-0	inhibitor of IlmtI enzyme
PHENOLSULFONPHTHALEIN	PHENOL RED	143-74-8	diagnostic aid [renal]
PHENOTHIAZINE	NEMAZINE	92-94-2	anthelmintic
PHENOXYBENZAMINE HYDROCHLORIDE	DIBENZYLINE	63-92-3, 59-96-1 [phenoxybenzamine]	alpha adrenergic blocker
PHENYL AMINOSALICYLATE	PHENYPASTEBAMIN	133-11-9	antibacterial (tuberculostatic)
PHENYLMERCURIC ACETATE	AGROSAN	62-38-4	antifungal, antimicrobial
PICROPODOPHYLLIN		477-47-4	Insulin growth factor 1 receptor inhibitor, antineoplastic
PIMETHIXENE MALEATE		314-03-4	H1 antihistamine
PIMOBENDAN	VETMEDIN	74150-27-9	PDE3 inhibitor, vasodilator, cardiotonic
PIMOZIDE	ORAP	2062-78-4	antipsychotic
PIPAMPERONE	DIPIPERON	1893-33-0	antipsychotic
PIZOTYLINE MALATE	SANDOMIGRAN	5189-11-7	5HT antagonist, antimigraine
POMIFERIN	POMIFITRIN	572-03-2	antioxidant, renal protectant
POMIFERIN DIMETHYL ETHER			
POMIFERIN TRIACETATE			
PREGNENOLONE SUCCINATE	FORMULA-405	4598-67-8	glucocortcoid, antiinflammatory
PROBUCOL	LORELCO	23288-49-5	antihyperlipidemic
PROCAINE HYDROCHLORIDE	NOVOCAIN	51-05-8, 59-46-1 [procaine]	anesthetic (local)

PROCHLORPERAZINE EDISYLATE	COMPAZINE	1257-78-9, 84-02-6 [prochlorperazine maleate], 58-38-8 [prochlorperazine]	antiemetic, antipsychotic, treatment of vertigo
PROCYCLIDINE HYDROCHLORIDE	KEMADRIN	1508-76-5, 77-37-2 [procyclidine]	anticholinergic
PROFLAVINE HEMISULFATE		553-30-0, 1811-28-5	antiseptic
PROMETHAZINE HYDROCHLORIDE	PHENERGAN	58-33-3, 60-87-7 [promethazine]	antihistamine
PROPOXUR	BAYGON	114-26-1	insecticide
PROPYLTHIOURACIL	PROPACIL	51-52-5	antihyperthyroid
PTAEROXYLIN		14729-11-4	
PTEROSTILBENE		537-42-8	antifungal
PURPURIN		81-54-9	xanthin oxidase inhibitor, irritant
PYRITINOL		1098-97-1	cognition enhancer, nootropic
PYRONARIDINE TETRAPHOSPHATE		76748-86-2	antimalarial
PYRVINIUM PAMOATE	POVAN	3546-41-6	anthelmintic, androgen receptor blocker
QUERCETIN		117-39-5, 6151-25-3(hydrate)	capillary protectant, antioxidant. antineoplastic, anti-HIV
QUINALIZARIN		81-61-8	antiviral, HIV-1 integrase inhibitor
RACECADOTRIL	TIORFAN	81110-73-8	antidiarrheal
RAFOXANIDE	FLUKANIDE	22662-39-1	anthelmintic
RALOXIFENE HYDROCHLORIDE	EVISTA	82640-04-8	antiestrogen
RAMIPRIL	ALTACE	87333-19-5	antihypertensive, ACE inhibitor
RESORCINOL MONOACETATE	EURESOL	102-29-4	antiseborrheic, antipruritic
RETINYL PALMITATE		79-81-2	provitamin, antixerophthalamic
RHODOCLADONIC ACID		26984-15-6	
RIFAPENTINE	PRIFTIN	61379-65-5	antibacterial
RIFAXIMIN	XIFAXAN	80621-81-4	antibacterial, RNA synthesis inhibitor
RITONAVIR	NORVIR	155213-67-5	antiviral
ROBENIDINE HYDROCHLORIDE		25875-50-7	coccidiostat
ROTENONE		83-79-4	acaricide, ectoparasiticide, antineoplastic, mitochondrial poison
ROTENONIC ACID, METHYL ETHER			
SALICYLANILIDE	SALINIDOL	87-17-2	antipyretic, fungicide
SANGUINARIUM CHLORIDE	VIADENT	5578-73-4	antineoplastic, antiplaque agent
SECURININE		5610-40-2	GABAA receptor blocker, CNS stimulant
SELENOMETHIONINE		1464-42-2	contrast agent
SENNOSIDE A	GLYSENNID	81-27-6	cathartic
SODIUM TETRADECYL SULFATE	SOTRADECOL	139-88-8	sclerosing agent
STRYCHNINE METHIODIDE			neuromuscular blocker
SULCONAZOLE NITRATE	EXELDERM	61318-91-0, 61318-90-9 [sulconazole]	antifungal
SULFISOXAZOLE ACETYL	GANTRISIN	80-74-0	antibacterial
SURAMIN HEXASODIUM	ANTRYPOL	129-46-4, 145-63-1 [suramin]	antiprotozoal, trypanocidal, antiviral
TACRINE HYDROCHLORIDE	COGNEX	1684-40-8	anticholinesterase, cognitive adjuvant, K channel blocker
TANNIC ACID		1401-55-4	nonspecific enzyme/receptor blocker
TANSHINONE IIA SULFONATE SODIUM			free radical scavenger
TAPENTADOL HYDROCHLORIDE	NUCYNTA	175591-23-8	analgesic, norepinephrine uptake blocker, mu-opiod receptor agonist

TEGASEROD MALEATE	ZELNORM	189188-57-6	5HT4 receptor agonist, peristaltic stimulant
TELMISARTAN	MICARDIS	144701-48-4	antihypertensive, angiotensin II blocker
TENIPOSIDE	VUMON	29767-20-2	antineoplastic
TENOXICAM	ALGANEX	59804-37-4	antiinflammatory
TEPOXALIN	ZUBRIN	103475-41-8	antipsoriatic
TERCONAZOLE	TERAZOL	67915-31-5	antifungal
TERFENADINE	SELDANE	50679-08-8	H1 antihistamine, nonsedating
TETRAMIZOLE HYDROCHLORIDE	ANTHELVET	5086-74-8	anthelmintic
THEAFLAVIN MONOGALLATES			
THIMEROSAL	MERTHIOLATE	54-64-8	antiinfective, preservative
THIOCTIC ACID	BILETAN	1077-24-7	hepatoprotectant
THIOGUANINE	TABLOID	154-42-7, 5580-03-0 [hemihydrate]	antineoplastic, purine antimetabolite
THIRAM	REZIFILM	137-26-8	antifungal
THYMOQUINONE		490-91-5	antineoplastic, antioxidant
TIOGUANINE	LANVIS	154-42-7	antineoplastic, anti-ulcerative colitis
TIOXOLONE	STEPIN	4991-65-5	antiseborrhoic
TOLNAFTATE	TINACTIN	2398-96-1	antifungal
TOLONIUM CHLORIDE	TOLAZUL	92-31-9	hemostatic
TOREMIFENE CITRATE	FARESTON	89778-27-8	antineoplastic, anti-estrogen
TORSEMIDE	DEMADEX	56211-40-6	diuretic, inhibits Na/K/2Cl carrier system
TOSUFLOXACIN TOLUENESULFONATE HYDRATE	TOSUXACIN	115964-29-9	antibacterial
TOTAROL		511-15-9	antimicrobial, neuroprotective
TRIFLUOPERAZINE HYDROCHLORIDE	STELAZINE	440-17-5	antipsychotic
TRIOXSALEN	TRISORALEN	3902-71-4	melanizing agent, antipsoriatic
TUBOCURARINE CHLORIDE PENTAHYDRATE	TUBARINE	6989-98-6, 57-94-3 [anhydrous], 41354-45-4 [replaced], 57-95-4 [tubocurarine]	muscle relaxant (skeletal)
URSOLIC ACID		77-52-1	diuretic, antineoplastic, antiulcer
UTILIN		31218-22-1	
VALACYCLOVIR HYDROCHLORIDE	VALTREX	124832-27-5	antiviral
VORTIOXETINE HYDROBROMIDE	BRINTELLIX	508233-74-7	antidepressant

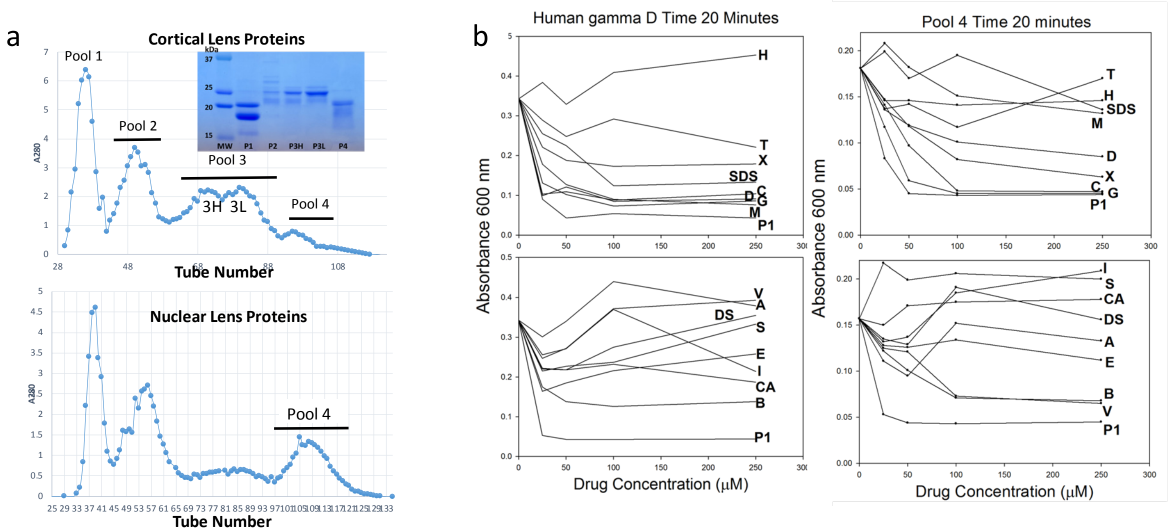

Fig. S1. **a**) Representative Sephadex G-200 gel filtration profile of bovine lens extract fractionated into 4 pools each corresponding to HMW-α-crystallins (Pool 1), LMW-α-crystallins (Pool 2), βH- and βL-crystallins and γ-crystallins (Pool 4) according to Ecroyd et al., respectively. **b**) Effect of concentration on the ability of candidate drugs Hematoporphyrin (H), Tetrasodium sulfate (T), Hexachlorophene (X), Avocadene (V), Chaulmoogric acid (M), Bixin (B), Closantel (C), Gambogic acid (G), Dihydrogambogic acid (D), Chaulmoogric acid (M), Sodium Dodecyl sulfate (SDS), Docusate sodium (DS), Sennoside A (S), Escin (E), Hematein (I), Citric Acid (CA), Bixin (B) and Bovine α-crystallin-rich (Pool 1) to inhibit thermal induced aggregation of recombinant human γD crystallin (WT-HγD) and bovine γ-crystallin-rich Pool 4 (1.5 mg/ml). For comparison with Pool 1 (α-crystallins) as positive control Pool 1 was tested at 0, 0.25: 1, 0.5:1, 1:1 and 2:1 Pool 1 : Pool 4 molar ratio. This data at highest suppression was used to rank the top seven drugs by efficacy of suppression of thermal induced turbidity (see Fig.1f and Fig.1g)

Ref: Ecroyd H, Garvey M, Thorn DC, Gerrard JA, Carver JA Amyloid fibrils from readily available sources: milk casein and lens crystallin proteins. Methods Mol Biol. 2013; 996: 103-17. doi: 10.1007/978-1-62703-354-1_6.

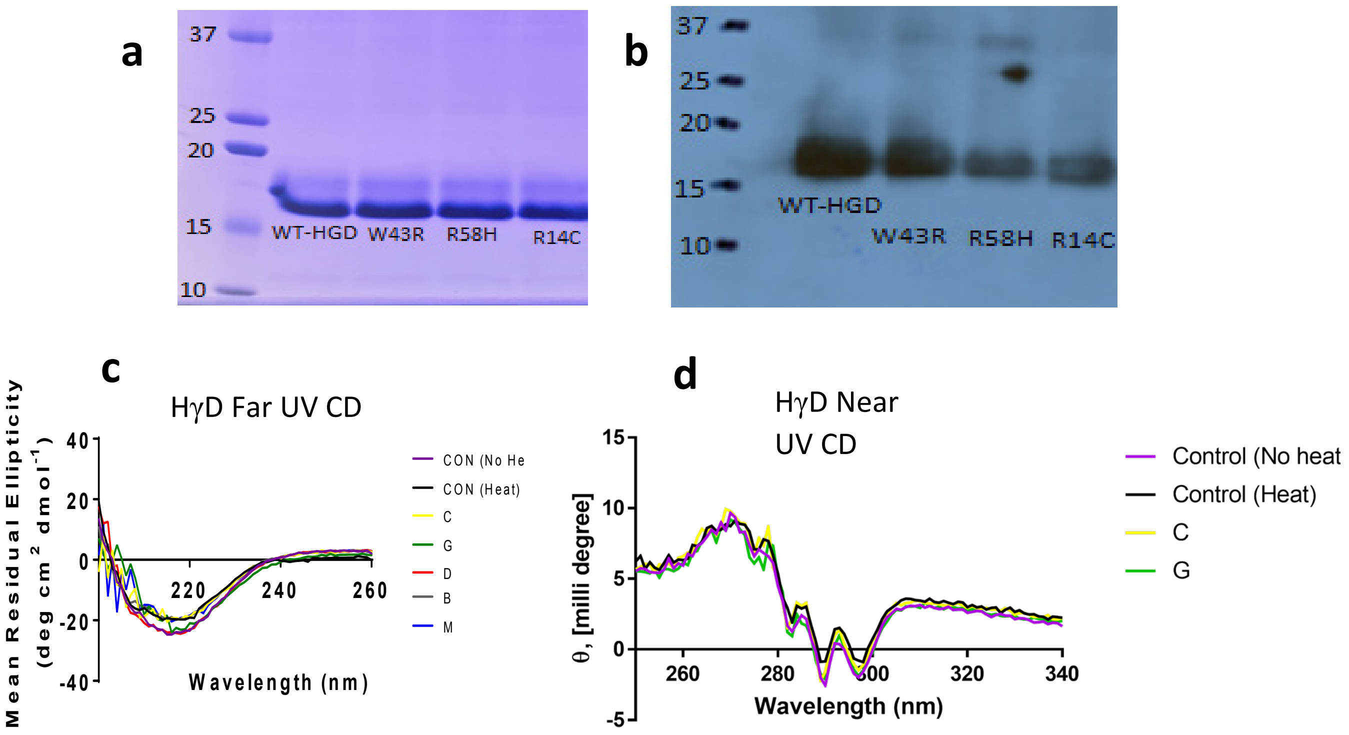

Fig. S2. **a)** SDS-Page of purified WT-HγD and its mutants (W43R, R58H, R14C) (20 μM protein in 50 mM $K^+PO_4^{3-}$) **b)** Western blot analysis of purified WT-HγD and its mutants (W43R, R58H, R14C) using anti-WT-HγD primary antibody at 1:5000 dilution. **c)** Far-UV CD spectra of WT-HγD without heat, with heat (72 °C, 20 min) alone and with drugs C, G, D, B, M. WT-HγD and drug concentrations used were 10 μM and 100 μM respectively in 10 mM sodium phosphate (pH 7.2). **d**) Near-UV spectra of WT-HγD without heat, with heat (72 °C, 20 min) alone and with drugs C and G. Overall the CD results indicate little to no changes in secondary or tertiary structure from heating WT-HγD for 20 min at 72 ºC.

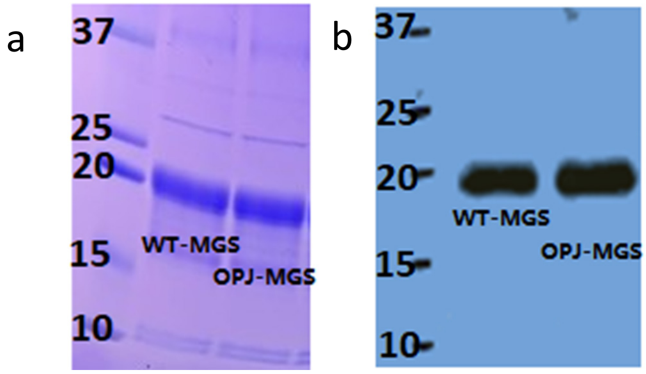

Fig S3. a) SDS-PAGE and western blot of WT-MγS (first lane) and its mutant OPJ-MγS (second lane) (20 μM in 50 mM $K^+PO_4^{3-}$). **b**) The western blot of WT-MγS and its mutant OPJ-MγS was done using anti-WT-MγS primary antibody at 1:5000 dilution.

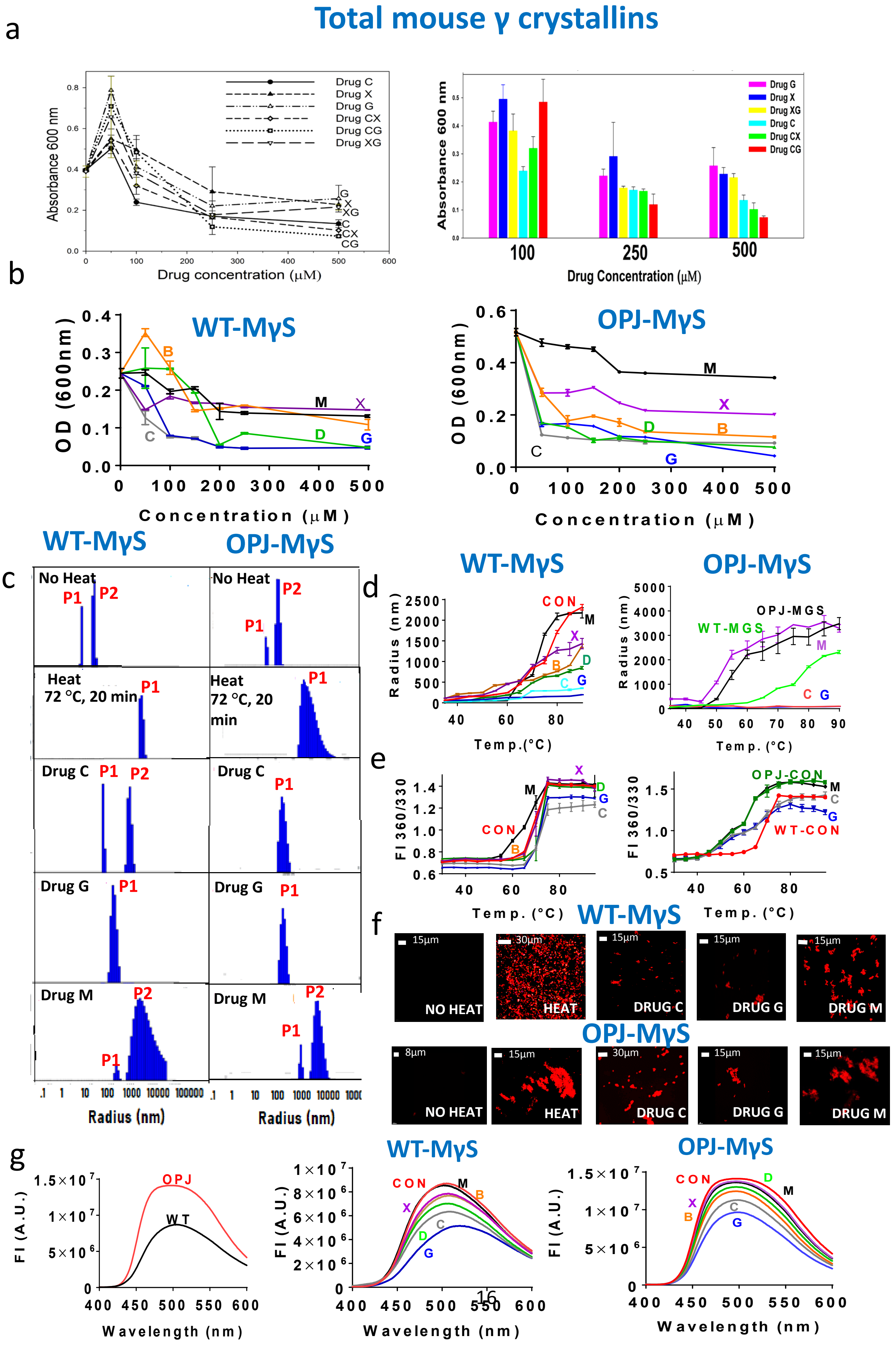
a
Total mouse γ crystallins
Absorbance 600 nm
Drug concentration (μM)
Drug C
Drug X
Drug G
Drug CX
Drug CG
Drug XG
G
X
XG
C
CX
CG
Drug G
Drug X
Drug XG
Drug C
Drug CX
Drug CG
100
250
500
Drug Concentration (μM)
b
WT-MγS
OPJ-MγS
OD (600nm)
Concentration (μM)
M
X
B
D
G
C
c
No Heat
Heat
72 °C, 20 min
Drug C
Drug G
Drug M
P1
P2
Radius (nm)
d
Radius (nm)
Temp.(°C)
CON
OPJ-MGS
WT-MGS
e
FI 360/330
OPJ-CON
WT-CON
f
NO HEAT
HEAT
DRUG C
DRUG G
DRUG M
15μm
30μm
8μm
g
FI (A.U.)
Wavelength (nm)
OPJ
WT

Fig. S4: Effects of Closantel, Gambogic acid and other drugs on the biophysical and morphological properties of total mouse gamma crystallins, WT-MγS and its mutant OPJ-MγS exposed to oxidative and thermal stress. **a)** Effect of various concentrations of drugs C, G, X and their combinations against H_2O_2 mediated oxidation (left panel) and thermal aggregation (72 °C, 20 min) (right panel) against total mouse gamma crystallins (2.5 mg/ml). **b)** Efficacy of drug C, G, C, D, B and M (0 µM, 50 µM, 100 µM, 150 µM, 200 µM, 250 µM, 500 µM) in preventing heat mediated aggregation (72 °C, 20 min) of WT-MγS (left panel) and OPJ-MγS (right panel) (45 µM each) (Note: mean±SD n=6 for 0µM drug, n=2 for all others). **c)** DLS spectra of thermally stressed samples of WT-MγS (left panel) and OPJ-MγS (right panel) (45 µM each) alone and with drug C, G and M (400 µM). **d)** DLS temperature scan WT-MγS (left panel) and OPJ-MγS (right panel) alone and with drugs (400 µM). **e)** Thermal unfolding assay of WT-MγS (left panel) and OPJ-MγS (right panel) (10 µM each) alone and with drugs (100 µM in DMSO). **f)** Fluorescence microscopic images of thermally stressed samples (72 °C, 20 min) of WT-MγS (upper panel) and OPJ-MγS (lower panel) (50 µM of each) alone and with drug C, G and M (400 µM) using Nile red. g, bis-ANS binding spectra of 10 µM of WT-MγS and OPJ-MγS alone (left panel), WT-MγS with drug C, G, X, D, B, M (100 µM each) (middle panel) and OPJ-MγS with drug C, G, X, D, B, M (100 µM) (right panel). The buffer was 50 mM K_3PO_4 buffer, pH 7.2. All the experiments were performed in triplicates. The results are mean±std of three individual readings, except for 3b).

HγD

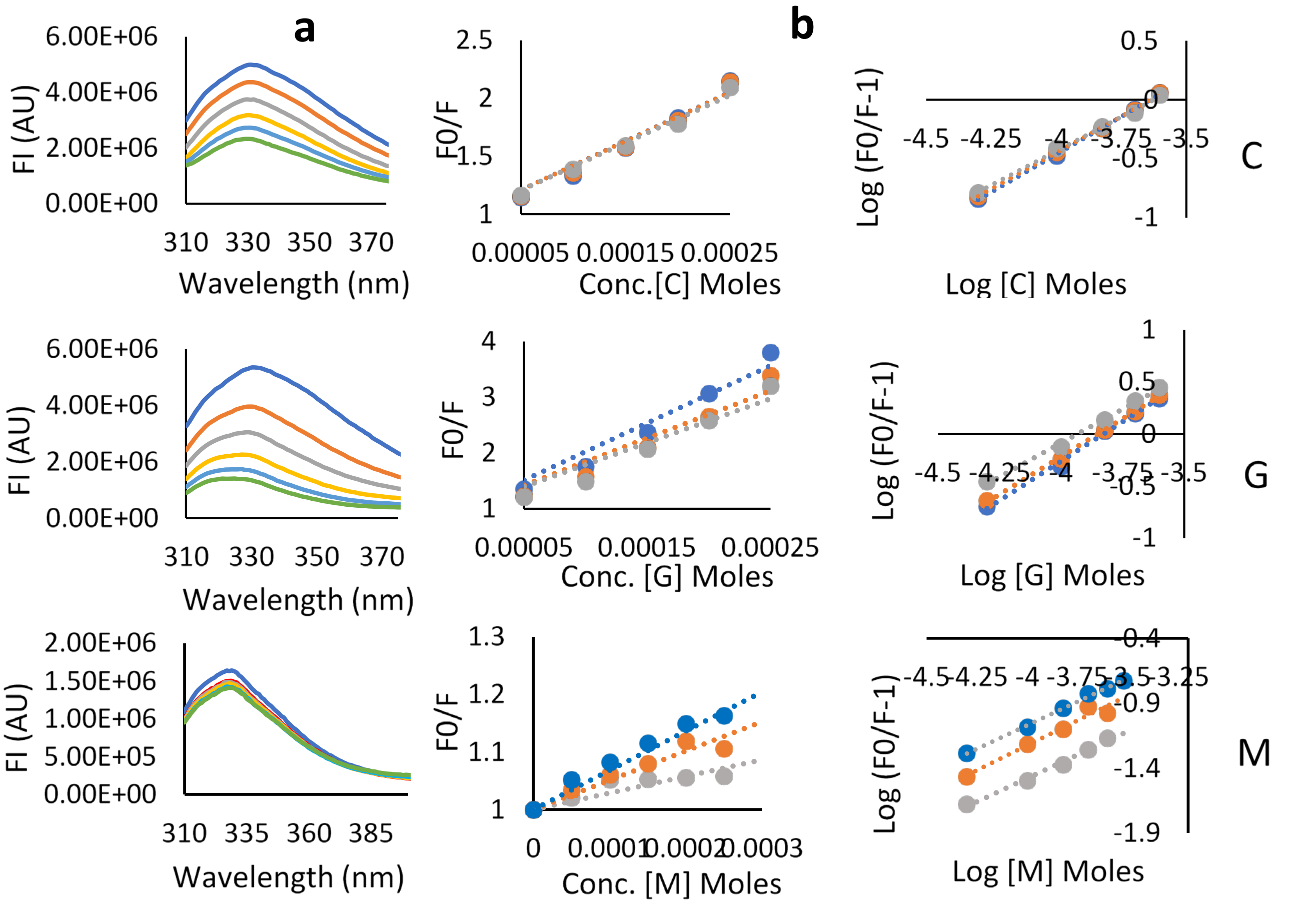

Fig. S5. **a)** Fluorescence emission spectra, **b)** Stern-Volmer plot and **c)** modified Stern-Volmer plot of tryptophan fluorescence quenching of WT-HγD (10 µM in 50 mM $K^+PO_4^{3-}$; pH 7.2) by increasing concentrations of drug C, G and M (0 µM: dark blue, 50 µM: red, 100 uM: grey, 150 µM: yellow, 200 uM: light blue, 250 µM: green). Fluorescence emission spectra was recorded at 25 °C, 30 °C and 37 °C. However, spectra of only 25 °C is shown here. Stern-Volmer and modified Stern-Volmer plots at 25 °C, 30 °C and 37 °C are indicated by blue, red and grey lines respectively.

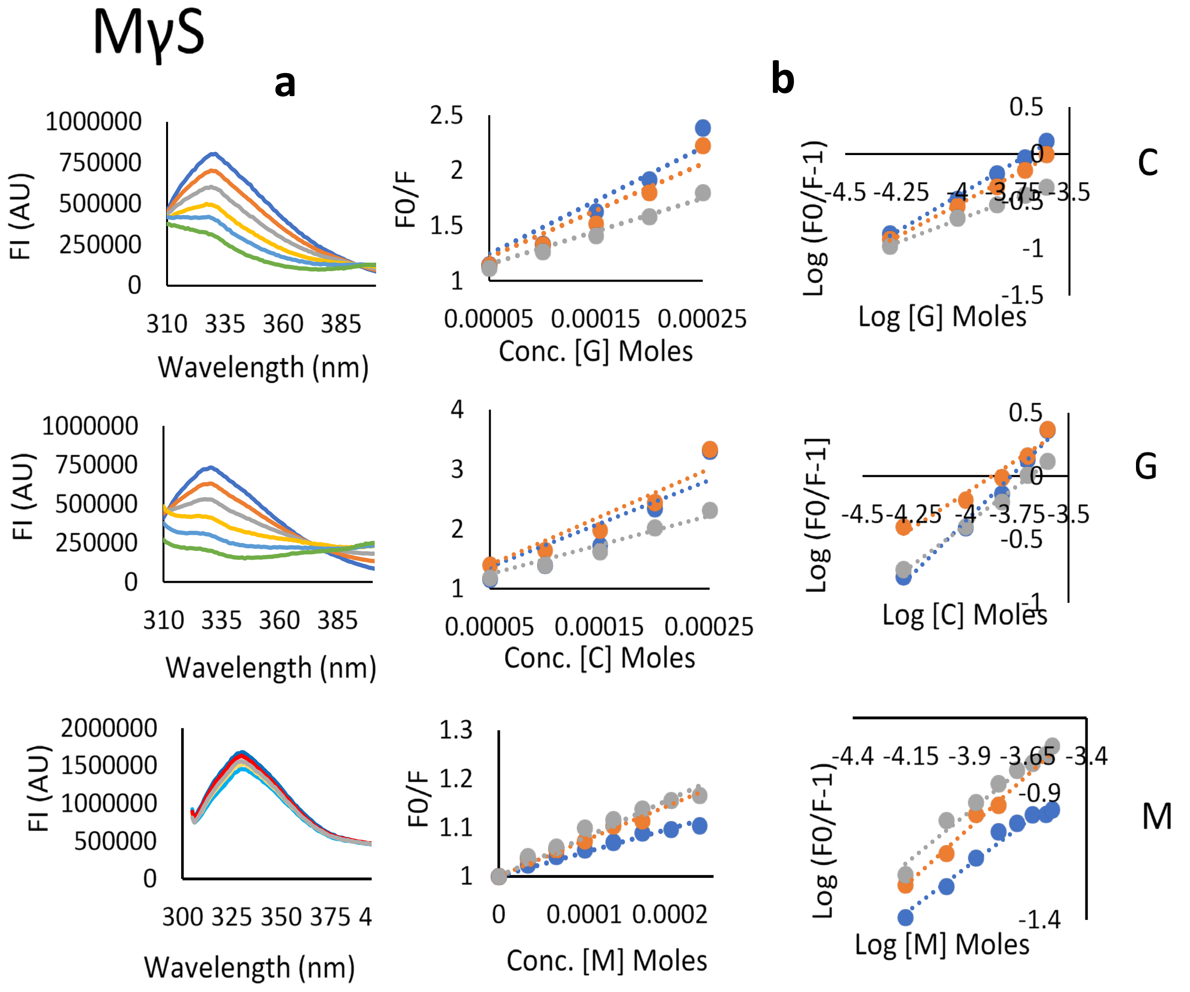

Fig. S6. **a)** Fluorescence emission spectra, **b)** Stern-Volmer plot and **c)** modified Stern-Volmer plot of tryptophan fluorescence quenching of WT-MγS (10 uM in 50 mM $K^+PO_4^{3-}$; pH 7.2) by increasing concentrations of drug C, G and M (0 µM: dark blue, 50 µM: red, 100 µM: grey, 150 µM: yellow, 200 µM: light blue, 250 µM: green). Fluorescence emission spectra was recorded at 25 °C, 30 °C and 37 °C. However, spectra of only 25 °C is shown here. Stern-Volmer and modified Stern-Volmer plots at 25 °C, 30 °C and 37 °C are indicated by blue, red and grey lines respectively.

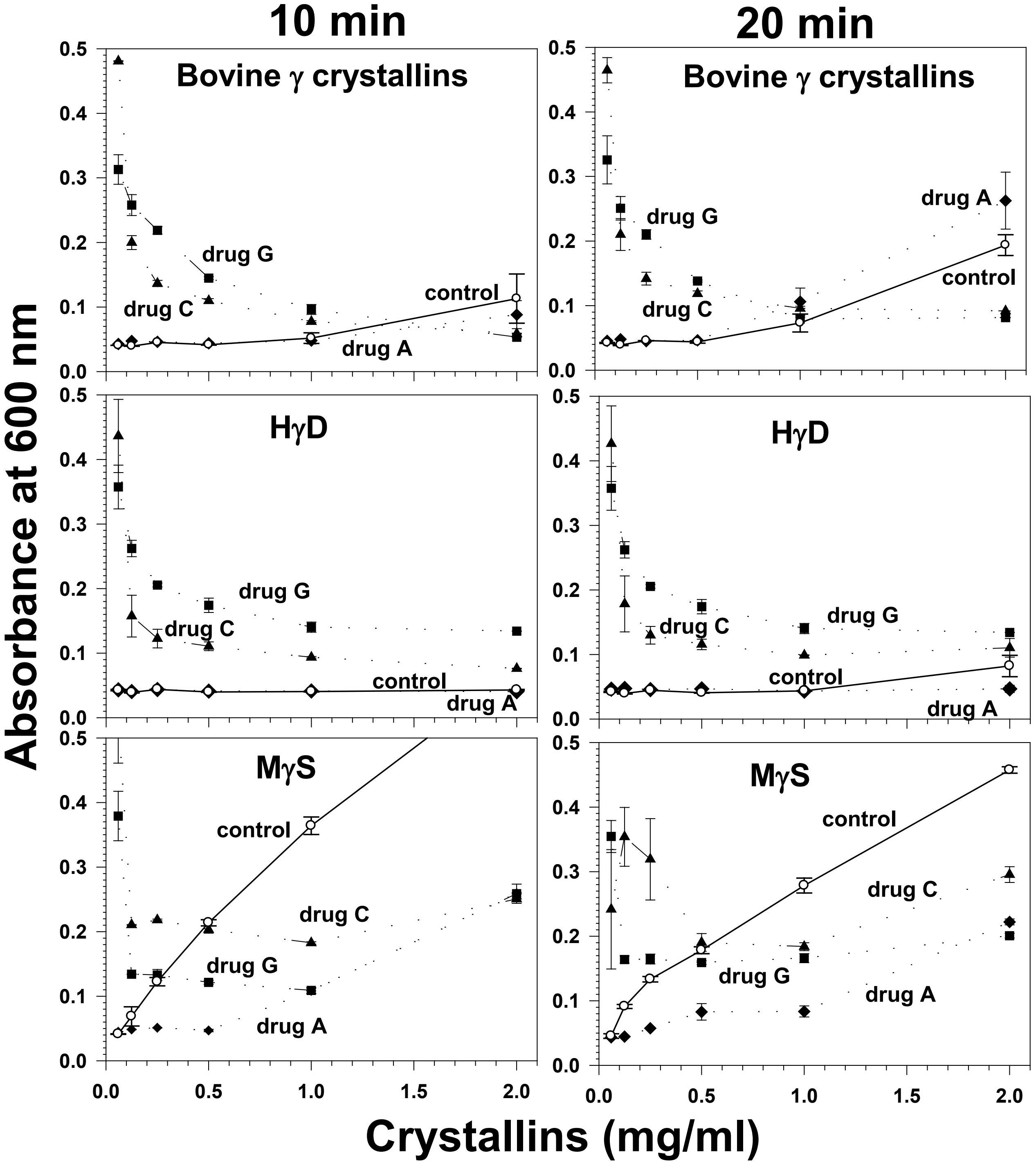

Fig. S7. Role of the protein concentration on crystallin turbidity measured at 600nm and its potential suppression by Drugs C, G and A. Total bovine gamma crystallins (BGC) (Pool 4), HγD and MγS were incubated at protein concentrations varying from 0.06 mg/ml to 2.0mg/ml Buffer A, i.e. 50 mM K+/PO4, pH 7.2) with no or 400 μM Drug C and G and A (Agaric Acid) in 5% DMSO. Microtiter plates were incubated first for 10 min at 72°C and re-incubated for 10 min. Absorbance was determined at both 10 and 20 min total incubation time. For full details and discussion see paragraph on Rigor and Reproducibility at the end of the main text.
